# Metabolism of Deoxynivalenol and Deepoxy-Deoxynivalenol in Broiler Chickens, Pullets, Roosters and Turkeys

**DOI:** 10.3390/toxins7114706

**Published:** 2015-11-12

**Authors:** Heidi E. Schwartz-Zimmermann, Philipp Fruhmann, Sven Dänicke, Gerlinde Wiesenberger, Sylvia Caha, Julia Weber, Franz Berthiller

**Affiliations:** 1 Christian Doppler Laboratory for Mycotoxin Metabolism, Center for Analytical Chemistry, Department of Agrobiotechnology (IFA-Tulln), University of Natural Resources and Life Sciences, Vienna, Tulln 3430, Austria; E-Mails: sylvia.caha@boku.ac.at (S.C.); franz.berthiller@boku.ac.at (F.B.); 2 Institute of Applied Synthetic Chemistry, Vienna University of Technology, Vienna 4060, Austria; E-Mails: philipp.fruhmann@tuwien.ac.at (P.F.); julia.weber@tuwien.ac.at (J.W.); 3 Centre of Electrochemical Surface Technology, Wiener Neustadt 2700, Austria; 4 Institute of Animal Nutrition, Friedrich-Loeffler-Institute (FLI), Federal Research Institute for Animal Health, Braunschweig 38116, Germany; E-Mail: sven.daenicke@fli.bund.de; 5 Department of Applied Genetics and Cell Biology, University of Natural Resources and Life Sciences, Vienna, Tulln 3430, Austria; E-Mail: gerlinde.wiesenberger@boku.ac.at

**Keywords:** mycotoxins, biomarkers, HPLC-MS/MS, poultry, deoxynivalenol-3-sulfate, deoxynivalenol sulfonates

## Abstract

Recently, deoxynivalenol-3-sulfate (DON-3-sulfate) was proposed as a major DON metabolite in poultry. In the present work, the first LC-MS/MS based method for determination of DON-3-sulfate, deepoxy-DON-3-sulfate (DOM-3-sulfate), DON, DOM, DON sulfonates 1, 2, 3, and DOM sulfonate 2 in excreta samples of chickens and turkeys was developed and validated. To this end, DOM-3-sulfate was chemically synthesized and characterized by NMR and LC-HR-MS/MS measurements. Application of the method to excreta and chyme samples of four feeding trials with turkeys, chickens, pullets, and roosters confirmed DON-3-sulfate as the major DON metabolite in all poultry species studied. Analogously to DON-3-sulfate, DOM-3-sulfate was formed after oral administration of DOM both in turkeys and in chickens. In addition, pullets and roosters metabolized DON into DOM-3-sulfate. *In vitro* transcription/translation assays revealed DOM-3-sulfate to be 2000 times less toxic on the ribosome than DON. Biological recoveries of DON and DOM orally administered to broiler chickens, turkeys, and pullets were 74%–106% (chickens), 51%–72% (roosters), and 131%–151% (pullets). In pullets, DON-3-sulfate concentrations increased from jejunum chyme samples to excreta samples by a factor of 60. This result, put into context with earlier studies, indicates fast and efficient absorption of DON between crop and jejunum, conversion to DON-3-sulfate in intestinal mucosa, liver, and possibly kidney, and rapid elimination into excreta via bile and urine.

## 1. Introduction

After being discovered in 1972 [1], the Fusarium mycotoxin deoxynivalenol (DON) is one of the most common contaminants of cereal-based food and feed. Since its discovery, a lot of research has been carried out on its occurrence [2,3], toxicity [4], and reduction [5,6] as well as on its metabolization by plants [7], humans [8], and animals [9]. Nevertheless, the current knowledge, particularly on metabolization by different animal species, is far from complete.

One metabolization type common to most studied animal species is de-epoxidation. Formation of deepoxy-DON (DOM) is predominantly achieved by the gut microbiota. Its extent varies greatly both between species and also between individuals of one species [9,10]. The second metabolization pathway of DON observed in most of the investigated animal species is glucuronidation. Glucuronidation is carried out by endogenous UDP-glucuronosyltransferases in liver, and possibly also in intestinal microsomes [11]. In general, the extent of glucuronidation and the regiospecificity of the reaction are species-dependent with additional great individual differences [11,12,13].

Both de-epoxidation and glucuronidation have been studied as metabolization pathways of DON for many years. In humans and pigs, de-epoxidation is of minor importance [14,15,16,17,18], whereas glucuronidation is a major metabolization step [8,14,18,19,20]. In ruminants [21,22] and in rats [23], both de-epoxidation and glucuronidation of DON and of the formed DOM are significant metabolic processes. In addition, very recently two independent research groups discovered sulfonation as the main metabolization pathway of DON in rats [24,25]. Poultry, however, shows only minor metabolization of DON by glucuronidation, de-epoxidation, and sulfonation [26]. In contrast, sulfation was recently discovered as a major metabolization pathway, with DON-3α-sulfate as the predominant metabolic product of DON in chickens and turkeys [25,26]. According to Wan and co-workers who used radioisotope counting radio-HPLC for quantitation, DON-3-sulfate in excreta of chickens accounted for 89% ± 7% of the administered dose of DON (2.5 mg/kg body weight (b.w.)) [25]. Very recently, the toxicokinetic behavior of DON after oral application to turkeys and broiler chickens was investigated, and the peak areas of DON-3-sulfate and DON in plasma were compared [26]. Five minutes after administration of DON, the DON-3-sulfate to DON ratio in plasma was already 38 for turkeys and almost 2000 for broiler chickens. Thirty minutes after treatment, ratios increased to 141 and to nearly 10,000 for turkeys and broilers, respectively. These data indicate rapid absorption as well as very rapid and extensive metabolization of DON to DON-3-sulfate in both investigated avian species.

Although DOM is a minor metabolite in poultry [27,28], de-epoxidation of DON-3-sulfate or rapid sulfation of DOM may still be significant processes. We therefore hypothesized that DOM-3-sulfate might equally be a natural DON metabolite in poultry. The first aim of our work was therefore to synthesize and characterize DOM-3-sulfate and DOM-15-sulfate and to investigate their toxicity on ribosomes, the molecular targets of trichothecenes. The second aim was to develop and validate the first LC-MS/MS-based method for simultaneous quantitation of DON, DOM, DON-, and DOM-sulfate as well as DON- and DOM-sulfonates (DONS and DOMS) in excreta of different poultry species. The third objective of the work was to prove the hypothesis of formation of DON-3-sulfate and DOM-3-sulfate, as well as of DONS and DOMS in poultry consuming feed contaminated with DON and/or DOM. To this end, excreta and, in one occasion, chyme samples from four previous feeding trials with turkeys, chickens, pullets [28], and roosters [29,30] were re-analyzed. In three of these trials, feed consumption was recorded and the biological recoveries of orally administered DON and DOM could be assessed. In addition, the effect of species (turkey and chicken) and concomitant infection with *Ascaridia galli* on DON metabolization could be investigated. The results of this work will contribute to the understanding of DON metabolization in poultry.

## 2. Results

### 2.1. Synthesis, Purification, and Characterization of DOM-3-Sulfate and DOM-15-Sulfate

**Figure 1:**
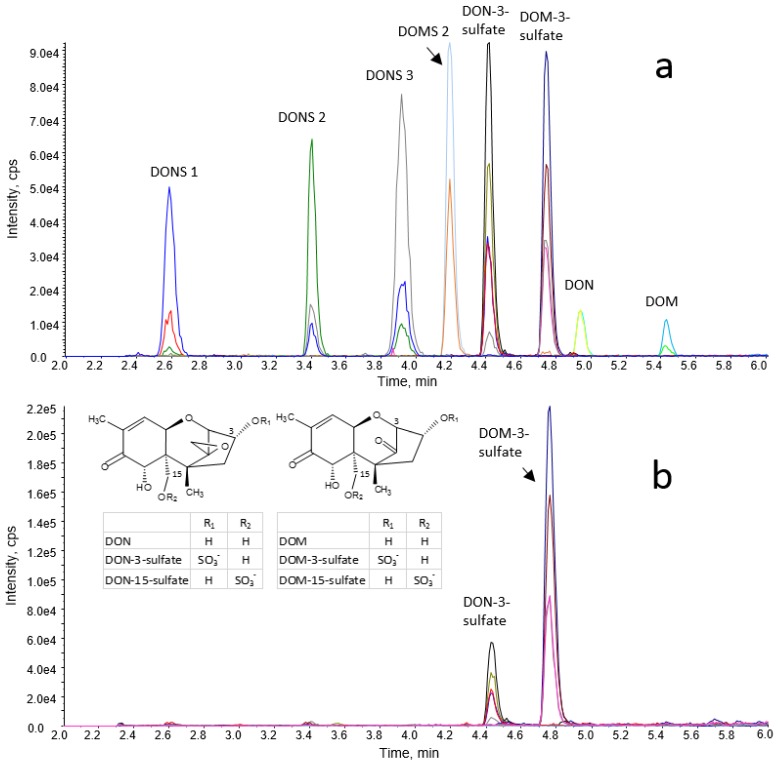
LC-MS/MS chromatogram of (**a**) a standard solution (30 ng/mL of DONS 1, DONS 2, DONS 3, DOMS 2, DON-3-sulfate, DOM-3-sulfate, DON, and DOM) and (**b**) of an excreta extract of a turkey fed a DOM-contaminated diet (1.6 mg/kg DOM and 0.2 mg/kg DON in feed).

Similarly to production of DON-sulfates [31], synthesis of DOM-sulfates is a two-step reaction. In the first step, three protected intermediates were produced: 2,2,2-trichloroethyl-DOM-3-sulfate (71.0 mg, 29%), 2,2,2-trichloroethyl-DOM-15-sulfate (14.0 mg, 6%) and, as by-product, bis(2,2,2-trichloroethyl) DOM-3,15-disulfate (30.0 mg, 9%). These substances were recovered as white solid with an overall yield of 44%. NMR data of the protected intermediates are given in the electronic supplementary material; NMR spectra are shown in Figure S1. Deprotection of the intermediates and column chromatography with ammonium hydroxide as a mobile phase additive yielded 36.7 mg (67% of the protected intermediate) of DOM-3-sulfate, 5.3 mg (49%) of DOM-15-sulfate (both as ammonium salt), and 9.2 mg (45%) of the by-product DOM-3,15-disulfate (as diammonium salt). By using LC-HR-MS, the following molecular masses were obtained. DOM-3-sulfate: 360.0880 g/mol (exact molecular mass: 360.0879 g/mol); DOM-15-sulfate: 360.0879 g/mol (exact molecular mass: 360.0879 g/mol); DOM-3,15-disulfate: 440.0448 g/mol (exact molecular mass: 440.0447 g/mol). Results of NMR measurements are summarized in the electronic supplementary material; NMR and LC-HR-MS/MS spectra are given in Figures S2 and S3. The structures of DON- and DOM-sulfates are provided in Figure 1.

### 2.2. Method Validation

For LC-MS/MS analysis, two LC gradient methods were developed, a long method capable of separating the 3/15 isomers of DON- and DOM-sulfate and a short method where the 3/15 isomers of DON- and DOM-sulfate are only partially separated. Chromatograms of a standard solution and of a turkey extract measured by the short method are shown in Figure 1, and a standard chromatogram recorded by the long method is depicted in Figure S4.

Prior to method validation, between four and six randomly selected samples of each matrix (excreta of turkeys, chickens, pullets, roosters, and chyme of pullets from both the DON- and DOM group, if available) were extracted and measured by the long gradient method. Due to presence of DON-3-sulfate and in part DOM-3-sulfate, but absence of the 15-sulfate isomer in all investigated samples, the long gradient method was validated only with respect to matrix effects, whereas the short gradient method was selected for full validation and later on for measurement of all samples.

Apparent recoveries (R_A_s), recoveries of extraction (R_E_s), and matrix effects (SSE) for the short routine method are summarized in Table 1. Signal suppression and enhancement (SSE) of the long gradient method are given in Table S1 in the Supplementary Material. Recoveries of extraction ranged between 87% and 96% for all analytes. Hence, apparent recoveries were mainly determined by mass spectrometric SSE. Matrix effects for DON and DOM were between 80% and 108% in all sample extracts, resulting in apparent recoveries between 71% and 107%. Matrix effects for DON-3-sulfate, DOM-3-sulfate, as well as for DON- and DOM sulfonates were mostly between 100% and 120% with the exception of all analytes in chyme sample extracts and DON- and DOM sulfonates in excreta extracts of chickens. Accordingly, R_A_s mostly ranged between 90% and 110%. However, matrix enhancement by approximately 150% was observed for DON-3-sulfate, DOM-3-sulfate, and DONS 1 in jejunum and ileum of pullets. Exceptionally large matrix enhancement occurred for DONS 2, DONS 3, and DOMS 2 in chyme sample extracts and additionally for DONS 3 in excreta extracts of chickens. One reason is the lower dilution factor for chyme samples compared to excreta extracts, which was required because of lower analyte concentrations in jejunum and ileum compared to excreta. Additionally, DON sulfonates and DON-3-sulfate exhibit severe matrix enhancement in cereal sample extracts [32,33], and chyme samples still contain greater proportions of undigested cereal matrix. As indicated by these data, quantitation of DON- and DOM- sulfates and sulfonates in chyme and excreta samples of poultry requires determination of matrix effects in appropriately diluted blank extracts and correction by the respective R_A_s.

**Table 1:** Validation of the short gradient method. R_A_: apparent recovery, R_E_: recovery of extraction, SSE: matrix effects.

		Dilution of Extract (v + v)	Average ± Standard Deviation (*n* = 3, Values in %)							
			DON-3-sulfate	DOM-3-sulfate	DON	DOM	DONS 1	DONS 2	DONS 3	DOMS 2
R_E_	Excreta of turkeys		90 ± 1	91 ± 1	91 ± 1	89 ± 1	90 ± 2	90 ± 3	91 ± 3	90 ± 2
	Excreta of chickens		86 ± 5	88 ± 3	93 ± 4	87 ± 3	91 ± 3	96 ± 3	87 ± 7	90 ± 3
SSE	Excreta of turkeys	1 + 2	102 ± 1	99 ± 0	96 ± 2	80 ± 2	103 ± 2	122 ± 3	105 ± 1	107 ± 1
	Excreta of chickens	1 + 2	117 ± 2	112 ± 3	117 ± 2	97 ± 1	121 ± 1	138 ± 1	208 ± 6	131 ± 1
	Jejunum of pullets	1 + 1	153 ± 1	150 ± 1	93 ± 1	107 ± 1	149 ± 2	229 ± 14	334 ± 25	242 ± 20
	Ileum of pullets	1 + 1	149 ± 7	138 ± 1	99 ± 1	107 ± 1	177 ± 2	292 ± 9	365 ± 9	256 ± 6
	Excreta of pullets	1 + 5	100 ± 1	110 ± 1	101 ± 0	101 ± 0	121 ± 3	115 ± 1	110 ± 2	115 ± 0
	Excreta of roosters	1 + 9	93 ± 0	110 ± 2	101 ± 1	108 ± 1	133 ± 2	116 ± 2	116 ± 0	107 ± 1
R_A_	Excreta of turkeys	1 + 2	92 ± 3	90 ± 2	87 ± 3	71 ± 3	92 ± 2	111 ± 1	96 ± 1	96 ± 2
	Excreta of chickens	1 + 2	98 ± 6	98 ± 6	107 ± 6	84 ± 4	110 ± 4	132 ± 7	178 ± 15	118 ± 2
	Jejunum of pullets	1 + 1	135 ± 2	134 ± 3	86 ± 2	94 ± 1	135 ± 1	213 ± 1	297 ± 5	218 ± 1
	Ileum of pullets	1 + 1	131 ± 1	124 ± 1	91 ± 1	94 ± 1	160 ± 2	272 ± 13	325 ± 22	230 ± 18
	Excreta of pullets	1 + 5	88 ± 6	98 ± 1	93 ± 1	89 ± 1	110 ± 2	107 ± 8	98 ± 8	104 ± 5
	Excreta of roosters	1 + 9	82 ± 1	98 ± 1	93 ± 0	95 ± 0	120 ± 3	108 ± 1	103 ± 2	96 ± 0

Limits of detection (LODs) and quantitation (LOQs) are given in Table 2. In pure standard solution, LODs ranged between 0.3 (sulfates and sulfonates of the series 2 and 3) and 1.5 ng/mL (DON, DOM), LOQs between 1.0 and 4.5 ng/mL. LODs and LOQs in lyophilized samples depended on the dilution factor of the extract. In chyme samples, LODs were between 40 (sulfates and sulfonates of the series 2) and 200 (DOM) ng/g, LOQs between 120 and 600 ng/g. Highest LODs (up to 1000 ng/g for DOM) and LOQs (up to 3000 ng/g) were obtained for excreta of roosters, the extract of which was diluted 1 + 9 (v + v) before measurement.

**Table 2:** Limits of detection (LODs) in pure standard solution and in freeze-dried chyme and excreta samples of poultry. Limits of quantitation (LOQs) were by the factor of 3.3 higher.

Matrix	DON-3-sulfate	DOM-3-sulfate	DON	DOM	DONS 1	DONS 2	DONS 3	DOMS 2
Pure standard solution (ng/mL)	0.3	0.3	1.5	1.5	0.6	0.3	0.3	0.3
Excreta of turkeys (ng/g)	59	98	196	300	119	59	68	59
Excreta of chickens (ng/g)	51	98	196	400	119	59	68	69
Jejunum of pullets (ng/g)	40	40	130	200	79	40	46	40
Ileum of pullets (ng/g)	40	40	130	200	79	40	46	40
Excreta of pullets (ng/g)	102	147	391	800	237	119	137	138
Excreta of roosters (ng/g)	198	245	652	1000	396	198	228	198

Calibration functions in neat standard solution were linear from the LOQ up to at least 674 ng/mL for all analytes. Matrix-matched calibration functions were linear in the investigated calibration ranges (up to 674 ng/mL for excreta extracts of turkeys and chickens; up to 600 ng/mL for chyme and excreta extracts of pullets and excreta extracts of roosters) with the following exceptions: calibration curves of DONS 2, DONS 3, and DOMS 2 were linear up to 300 ng/mL in chyme extracts, and calibration functions of DONS 3 were linear up to 337 ng/mL in excreta extracts of chickens.

The relative standard deviation (RSD) of triplicate work-up and measurement of blank turkey and chicken excreta samples spiked at 4 concentration levels on one day was below 10% for each substance at each concentration level (mostly between 2% and 5%). The inter-day RSD of work-up and analysis of one freeze-dried excreta sample of one turkey of the DOM group containing 834 ng/g DON-3-sulfate and 3420 ng/g DOM-3-sulfate was 11% and 9% for DON-3-sulfate and DOM-3-sulfate, respectively (*n* = 10). The inter-day RSD of sample preparation and measurement of one broiler excreta sample containing 7720 ng/g DON-3-sulfate that was worked-up and analysed on 10 different days was 5%.

### 2.3. Analysis of Samples

Excreta and chyme samples of four different feeding trials with different poultry species were carried out in the past in order to study the metabolization of DON and DOM, to assess biological recoveries, and to investigate the effects of DON on health and performance in poultry. These samples were re-analysed using the newly developed LC-MS/MS method, which included DON- and DOM-3-sulfate as well as DON- and DOM sulfonate metabolites. An overview of these four feeding trials is given in Table 5.

Turkey and chicken trial: The two feeding trials with turkeys and chickens were carried out in order to study the metabolization of DON and DOM in poultry and in order to assess the biological recoveries of these two mycotoxins after oral administration at concentrations relevant in practice. For that, the mycotoxin content in poultry feed (between 1.5 and 1.7 mg/kg) was kept well below the guidance value of 5 mg/kg in complementary and complete feeding stuffs. As birds excrete a mixture of white pasty urine and feces via the cloaca, only the combined excreta samples were collected.

**Figure 2:**
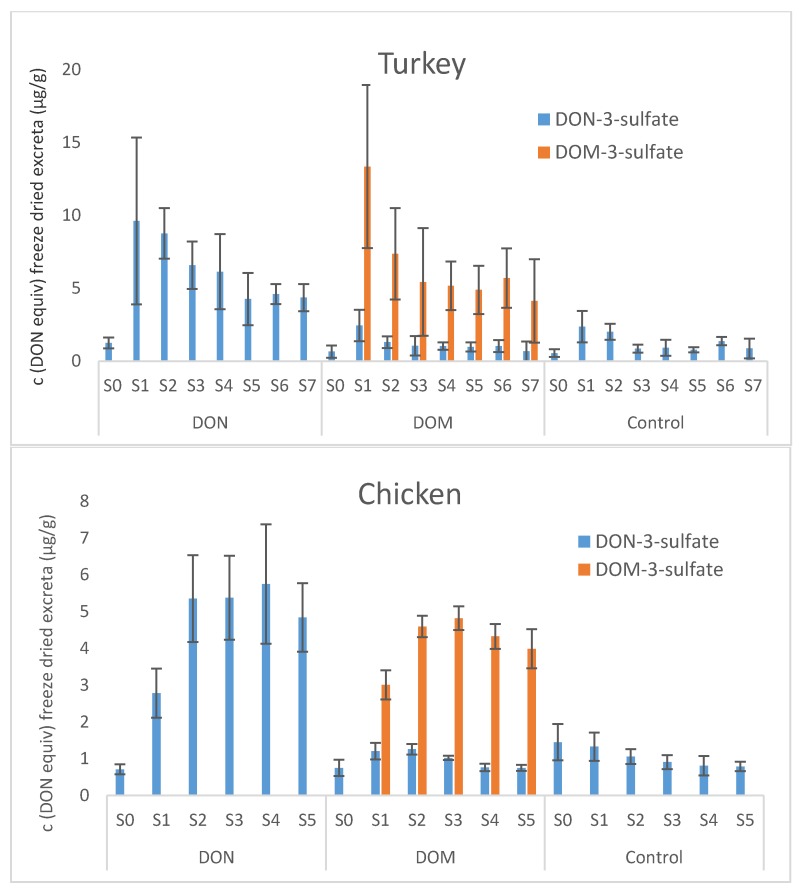
Average DON equivalent concentrations ± standard deviation (*n* = 4) of DON-3-sulfate and DOM-3-sulfate in excreta of turkeys and chickens at different sampling times (turkeys: 0, 2, 4, 6, 8, 11, 15 and 24 h after start of feeding; chickens: 0, 3, 6, 9, 12 and 24 h after start of feeding).

Both in turkeys and in chickens, DON-3-sulfate was the major DON metabolite, followed by DON. Analogous to that, DOM-3-sulfate turned out to be the major metabolite of orally administered DOM. DOM itself could not be detected in any of the excreta samples of the turkey and the chicken trial, nor could any of the DON- or DOM sulfonates. The average DON equivalent concentrations of the main DON- and DOM metabolites in excreta of turkeys and chickens (*n* = 4 for each species) at the individual sampling times are given in Figure 2. In lyophilized excreta of the individual turkeys of the DON group, maximum DON equivalent concentrations of DON-3-sulfate ranged from 8.8 to 16 μg/g and were obtained between 2 h (mean concentration 9.6 μg/g) and 4 h (mean concentration 8.8 μg/g) after provision of feed. Maximum DON equivalent concentrations of DOM-3-sulfate in excreta of turkeys of the DOM group were measured in excreta collected 2 h after start of feeding and ranged between 5.3 and 18 μg/g (average 13 μg/g). Due to the presence of 0.3 mg/kg DON in feed of the DOM group, DON-3-sulfate was also excreted by turkeys of the DOM group. The excretion pattern was similar to that of DOM-3-sulfate, with a maximum mean value of 2.5 μg/g in DON equivalents. A chromatogram of a turkey excreta extract of the DOM group is given in Figure 1.

In freeze-dried excreta of chickens, average DON equivalent concentrations of DON-3-sulfate in the DON group and of DOM-3-sulfate in the DOM group were 2.8 and 3.0 µg/g, respectively, 3 h after provision of feed. At the later sampling points (6, 9, 12 and 24 h after start of feeding), average DON equivalent concentrations of DON-3-sulfate increased to between 4.8 and 5.8 µg/g in the DON group and mean DON equivalent concentrations of DOM-3-sulfate were between 4.0 and 4.8 µg/g in the DOM group. The average DON equivalent concentration of DON-3-sulfate in excreta of the control group was between 0.8 and 1.3 µg/g.

The feed consumption by turkeys was not significantly different for the three feeding groups (DON, DOM, control) and ranged from 110 to 380 g per turkey on the day of the feeding trial. Likewise, feed consumption in chickens was similar across the three feeding groups with average values between 105 and 115 g per chicken and day. In Table 3, the average amounts of DON-3-sulfate, DOM-3-sulfate and DON excreted by turkeys and chickens between 2 h and 24 h after the start of feeding are given. Comparison of DON equivalent amounts of total excreted metabolites and ingested toxins yielded biological recoveries in turkeys between 34% and 71% (52% ± 16%, average ± standard deviation, *n* = 4) in the DON group, between 33% and 76% (51% ± 18%) in the DOM group, and between 68% and 75% (72% ± 3%) in the control group. In chickens, biological recoveries ranged between 69% and 95% (80% ± 13%) in the DON group, between 68 and 78% (74% ± 4%) in the DOM group, and between 93% and 126% (106% ± 14%) in the control group.

**Table 3:** Amounts of DON- and DOM metabolites excreted by turkeys and chickens between 2 h and 24 h after start of feeding (average ± natural standard deviation, *n* = 4, values in μg DON equivalents).

	Group	DON-3-sulfate	DOM-3-sulfate	DON	Sum Excreted	Consumed *	Biological Recovery (%)
Turkey	DON	144 ± 40	0.7 ± 0.8	5.2 ± 1.2	150 ± 42	304 ± 96	52 ± 16
	DOM	27 ± 13	149 ± 67	1.0 ± 1.4	177 ± 80	359 ± 159	51 ± 18
	Control	29 ± 7	0 ± 0	0 ± 0	29 ± 7	40 ± 10	72 ± 3
Chicken	DON	450 ± 112	0 ± 0	17 ± 12	476 ± 118	591 ± 77	80 ± 13
	DOM	72 ± 5	321 ± 30	1 ± 3	397 ± 33	536 ± 17	74 ± 4
	Control	71 ± 15	0 ± 0	0 ± 0	71 ± 15	67 ± 5	106 ± 14

* The average amounts of DON and DOM consumed were calculated by multiplying the concentration in feed by the amount of feed consumed by the individual animals between start and end of feeding.

Pullet trial: The original aim of the feeding trial with pullets was to investigate interactions between *Ascaridia galli* infection of pullets and DON contamination of feed [28]. As DON-3-sulfate and DOM-3-sulfate had not been suspected as major DON metabolites in poultry at that time, biological recoveries had been poor (≤5%). Therefore, the excreta samples obtained in this animal experiment were re-analysed by the newly developed LC-MS/MS method including DON- and DOM-sulfate and sulfonate metabolites. As contents of ileum and jejunum were also collected, the metabolite pattern across the pullets’ GI tract could be studied as well.

The average concentrations of DON metabolites in chyme and excreta samples are given in Table 4. Again, DON-3-sulfate was the main DON metabolite. However, contrary to chicken of the feeding trial described above, DOM-3-sulfate was also present in ileum and excreta samples of pullets. In addition, DON sulfonate metabolites of the series 1, 2 and 3 could be detected and quantified in jejunum and ileum samples. In general, both the metabolite concentrations and the metabolite pattern changed across the GI tract. In jejunum, DON-3-sulfate concentrations in the DON groups with and without *A. galli* infection were on average 3.5 times lower than in ileum and 60 times lower than in excreta samples. DOM-3-sulfate concentrations also increased from jejunum to excreta, reaching about 12%–15% of the DON-3-sulfate concentration in the latter matrix. DON sulfonate 2 (DONS 2) and DON sulfonate 3 (DONS 3) could be quantified in jejunum of the DON groups. In ileum, the concentration of DONS 2 remained similar, but DONS 3 could not be detected, most likely due to its instability under alkaline conditions [24,34]. However, traces of DONS 1 were observed in ileum, which might result from decomposition of DONS 3. In excreta, limits of quantitation were higher because of measurement of more diluted samples, so that only traces of DONS 2 were detected.

**Table 4:** Average concentrations of DON metabolites in chyme and excreta samples of pullets receiving 65 g feed containing 4.4 mg/kg DON (corresponding to 286 μg DON) per day. CON: negative control, -: no *A. galli* infection, +: *A. galli* infection, n.d.: not detected, tr: traces.

Matrix	Group	*n*	c (Average ± std dev, µg/g Freeze Dried Sample in DON Equivalents)					
			DON-3-Sulfate	DOM-3-Sulfate	DON	DONS 1	DONS 2	DONS 3
Jejunum	CON -	3	n.d.	n.d.	n.d.	n.d.	n.d.	n.d.
	CON +	3	tr *	n.d.	n.d.	n.d.	n.d.	n.d.
	DON -	3	0.34 ± 0.04	n.d.	tr	n.d.	0.20 ± 0.05	0.08 ± 0.02
	DON +	3	0.35 ± 0.04	n.d.	tr	n.d.	0.17 ± 0.00	0.12 ± 0.04
Ileum	CON -	2	tr	n.d.	n.d.	n.d.	n.d.	n.d.
	CON +	3	tr	n.d.	n.d.	n.d.	n.d.	n.d.
	DON - **	2	1.26 ± 0.53	tr	tr	tr	0.10 ± 0.01	n.d.
	DON +	3	1.09 ± 0.11	1.00 ± 1.38	tr	tr	0.20 ± 0.12	n.d.
Excreta	CON -	9	1.83 ± 0.34 ^a^	tr	n.d.	n.d.	n.d.	n.d.
	CON +	9	2.00 ± 1.00 ^a^	tr	n.d.	n.d.	n.d.	n.d.
	DON -	9	22.9 ± 0.8 ^b^	3.47 ± 0.14 ^a^	tr	n.d.	tr	n.d.
	DON +	9	20.3 ± 1.8 ^c^	2.49 ± 1.02 ^b^	tr	n.d.	tr	n.d.

*: tr in one sample of three. **: for groups with *n* = 2, the value is given as average ± (max-min)/2. Statistical analysis (excreta): mean values with different superscripts within one column are significantly different.

Biological recoveries reached values of 151% ± 5% in the DON group without *A. galli* infection and 131% ± 11% in the DON group infected by *A. galli* (see Table S2 in the Supplementary Material). Interestingly, statistical analysis revealed significant differences both between the concentrations and between the excreted amounts of DON-3-sulfate and DOM-3-sulfate in excreta of pullets of the DON groups with and without infection by *A. galli*. Excreta of pullets not infected by *A. galli* showed significantly greater concentrations of both sulfate metabolites than excreta of pullets infected with worms. Likewise, pullets not infected by *A. galli* excreted significantly greater amounts of DON-3-sulfate and of DOM-3-sulfate than pullets infected with *A. galli*.

Roosters: Excreta samples of roosters administered feed contaminated with 11 mg/kg DON [29] were analysed in order to study the metabolite pattern in roosters. Average DON-3-sulfate concentrations were 29 ± 3 μg/g freeze-dried excreta in DON equivalents. Surprisingly, mean DOM-3-sulfate concentrations amounted to 15 ± 3 μg/g in DON equivalents, yielding a DON-3-sulfate to DOM-3-sulfate ratio of 1.9 to 1. DONS 2 occurred at 0.8 ± 0.1 μg/g in DON equivalents, whereas only traces of DON were detected. Other compounds were below the respective LODs in rooster excrement extracts (see Table 2).

### 2.4. Toxicity assessment of DOM-3-Sulfate and DOM-15-Sulfate

**Figure 3:**
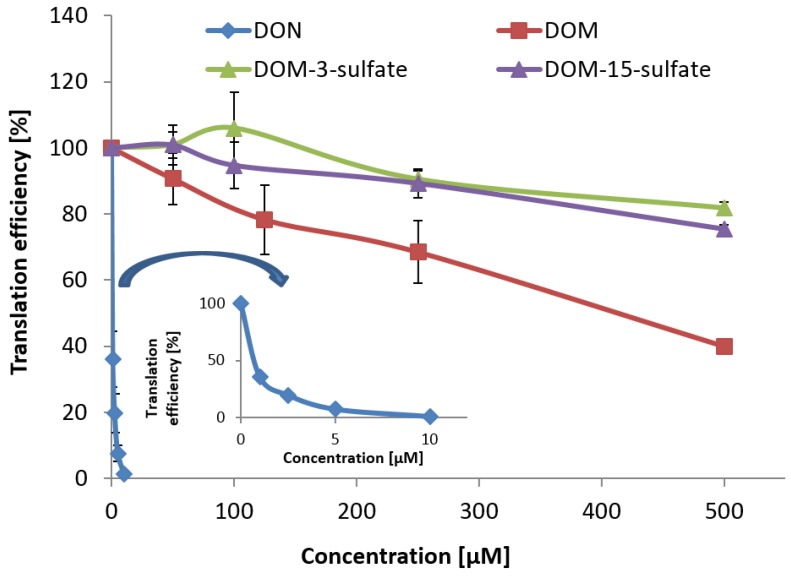
Comparative toxicity of DON, DOM, DOM-3-sulfate, and DOM-15-sulfate on the ribosome, determined by an *in vitro* transcription/translation assay with rabbit reticulocyte lysate. Error bars denote the analytical standard deviation of three independent determinations.

The toxicity of DON, DON-3-sulfate, and DON-15-sulfate on the ribosome had recently been assessed in *in vitro* transcription/translation assays with wheat germ extract [31]. In short, DON-3-sulfate did not show any toxicity in the investigated concentration range, whereas DON-15-sulfate was *ca*. 44 times less toxic than DON. To compare the toxicities of DON, DOM, DOM-3-sulfate, and DOM-15-sulfate, *in vitro* transcription/translation assays were performed with mammalian ribosomes (Figure 3). While 50% reduction of *in vitro* translation by rabbit reticulocyte lysate was obtained at 0.6 μM DON, the IC_50_ value of DOM was 410 μM. DOM-3-sulfate and DOM-15-sulfate did not significantly inhibit *in vitro* translation at concentrations up to 100 μM. The translation efficiency was still 81% and 75% at 500 μM DOM-3-sulfate and DOM-15-sulfate, respectively. Inhibition of translation was not significantly different for DOM-3-sulfate and DOM-15-sulfate at substrate concentrations up to 250 μM. At a concentration of 500 μM, differences in translation were minor, but significant. Based on IC_20_ values for DON (0.25 μM), DOM (122 μM), DOM-3-sulfate (490 μM), and DOM-15-sulfate (420 μM), DOM-3-sulfate is less toxic than DOM by a factor of four and less toxic than DON by almost a factor of 2000. Similarly, DOM-15-sulfate shows lower toxicity on the ribosome than DOM by a factor of 3.4 and lower toxicity than DON by a factor of nearly 1700. Statistical analysis revealed significantly greater inhibition of translation by DOM compared to DOM-3-sulfate and DOM-15-sulfate from 250 μM onwards. These results confirm that de-epoxidation of DON leads to a less toxic metabolite and that sulfation of trichothecenes is, similar to glucosylation in plants, a detoxification mechanism.

## 3. Discussion

Concluding from our results and those published in the most recent literature [25,26], DON-3-sulfate and DOM-3-sulfate are the missing link to quantitative biological recoveries of DON in poultry. Similar to previous studies reporting percentages of DON in excreta as being between 2% and 6% of total ingested DON [28,35], excreted DON was 2% and 3% of ingested DON in our turkey and chicken trial and approximately 4% in the pullet experiment. DOM was rarely detected in excreta samples in the literature. Whereas Wan *et al.* and Awad and co-workers could not detect DOM in excreta extracts [25,35], Dänicke *et al.*, applying immunoaffinity clean-up as sample preparation, determined 0.5 μg DON/kg b.w./day for pullets consuming *ca.* 300 μg DON/kg b.w./day, corresponding to approximately 0.2% of ingested DON [28]. Re-analyzing these pullet excreta samples, we could not detect DOM, most likely due to higher LODs due to greater dilution factors (see below). Similarly, DOM could not be detected in any of the excreta samples of our turkey and chicken experiments. One reason might be lack of microbes capable of de-epoxidizing DON. Another reason is that our methods were not designed to detect trace concentrations of DOM, but to allow co-determination of DON- and DOM-sulfates, DONS, DOMS, DON and DOM. Separation of DONS and DOMS requires formic acid as a mobile phase additive [24]. However, use of formic acid resulted in fivefold higher LODs for DON and DOM compared to acetic acid. Still, this compromise had to be made for proper separation of DON sulfonates. LOQ values for DON and DOM in a pure standard solution (5 ng/mL) were higher than those obtained by Devreese *et al.* (0.1 and 0.5 ng/mL for DON and DOM), but similar to those obtained in our previous work on DON- and DOM sulfonates (2 ng/mL on 5500 QTrap, 5 ng/mL on 4000 QTrap). LOQ values in lyophilized pullet feces obtained by Dänicke and co-workers were 0.8 and 1.6 ng/g [28], and as such far better than ours. The reason for this great difference is that Dänicke *et al.* used multi-step sample preparation including defatting and immunoaffinity clean-up. In our work, on the contrary, great dilution factors were chosen on purpose due to the high concentrations of DON-3-sulfate and in order to minimize matrix effects.

As DOM and DOM-sulfates are much less toxic than DON in an *in vitro* translation assay using animal ribosomes, and the main mode of action of all trichothecenes is the suppression of translation, these metabolites can be considered as detoxification products of DON. In the following, the question about location of formation, absorption and elimination of DON-3-sulfate and DOM-3-sulfate will be tackled by putting our findings and recent literature results in context with older studies on DON metabolization in poultry. For this purpose, it is important to note that poultry excrete white pasty urine into the cloaca where it is mixed with solid excrements from the GI tract. Hence, administration of native DON to poultry and collection of excrements does not give information about the origin of excreted DON-3-sulfate and DOM-3-sulfate. Another peculiarity of poultry is that, in addition to the hepatic portal system, a renal portal system exists. Both systems drain the intestine via the *Vena mesenterica cranialis* and *V. mesenterica caudalis*, respectively, and might contribute to renal and/or hepatic metabolism and first pass elimination as suggested by Rotter *et al.* [36].

As early as in 1986, Prelusky and co-workers administered a single oral dose of ^14^C-labeled DON to white Leghorn hens by crop intubation [37]. They observed low levels of radioactivity in plasma and reported the total body burden (excluding bile, GI tract and excreta) of radioactively-labeled DON and its metabolites to be less than 2%. The highest accumulation of radioactivity was determined in bile 6 h after treatment. Within 24 h, 79% of radioactivity was excreted. These results suggested low systemic absorption of DON and/or its metabolites and significant biliary excretion.

Two years later, Lun *et al.* [38], administering native DON to laying domestic chickens, reported that DON disappeared from the GI tract between the crop and the jejunum. However, only low levels of DON were detected in portal vein blood. Considering little decrease of DON when incubated with gastric fluid or juice from the small intestine [38], they set up the hypothesis of rapid post-absorptive modification of DON by enterocytes and hepatic elimination into bile. In the same study, *in vitro* incubation of native DON with contents of colon and caecae resulted in the reduction of the DON concentration by factors of 3, 9 and 20 after 6, 12 and 24 h of incubation, respectively.

One year later, Lun and co-workers administered tritium labeled DON by crop intubation to colostomized white Leghorn hens [39]. Interestingly, 68% of the administered radioactivity was excreted within 24 h into urine, whereas only 6% was eliminated via feces. Radioactivity in systemic blood accounted for only 7% of the administered radioactivity. Radioactivity along the GI tract of intact crop intubated hens decreased rapidly after the crop, but increased again in the large intestine six hours after administration, which is most likely due to retrograde movement of urine from cloaca into colon. However, contrary to observations by Prelusky and co-workers [37], biliary excretion of radioactivity was <1%. These results confirmed the hypothesis set up by the same working group in 1988 that ingested DON is rapidly absorbed between crop and upper jejunum and metabolized shortly after absorption [38]. Yet, extensive excretion of the altered DON via bile was not confirmed as most of the radioactivity was excreted into urine.

Results obtained in more recent studies where native or radiolabeled DON was administered to poultry mostly confirmed and complemented the earlier published data. Yunus *et al.* [27] recovered only 0.04% of total DON fed to broilers 1 h after oral treatment in plasma in the form of DON and DOM. Likewise, Dänicke and co-workers determined only trace levels of DON in plasma of pullets fed with poultry feed containing 4.4 mg/kg DON. However, two recent studies reported an oral bioavailability of 20% for DON administered to broiler chickens [40], and 21% for DON fed to turkeys [26]. Higher values than previously reported might be due to different ways of calculating the oral bioavailability. In accordance with earlier studies, rapid absorption, fast elimination, and extensive biotransformation were reported [26,40]. Comparison of oral and IV application of DON and inclusion of DON-3-sulfate in the analytical method for semiquantitative determination yielded precious information on the toxicokinetic behavior of DON and on the location of sulfation in turkeys and broiler chickens [26]. Already 5 min after oral administration, DON and DON-3-sulfate were detected in plasma. Maximum values of DON were reached 10 min post dosing, and maximum values of DON-3-sulfate between 20 and 30 min after application. Only 5 min after IV administration of DON, the average peak area ratio of DON-3-sulfate to DON was 10 in turkeys and 243 in broiler chickens. This finding points to very rapid sulfation, with the liver, extrahepatic tissues, and the intestinal mucosa being potential metabolism sites. In addition, average peak area ratios of DON-3-sulfate to DON were much greater after oral, rather than after IV administration in both avian species. This observation strongly substantiates the hypothesis by Lun *et al.* [38,39] that sulfation is already performed in enterocytes of the intestinal mucosa.

Our current turkey and chicken trial revealed that metabolization of DON and DOM was very similar in both avian species. Excretion kinetics could not be established because animals had *ad libitum* access to DON and DOM contaminated feed during the day. Despite large inter-individual differences between the turkeys of one feeding group, maximum metabolite concentrations in excreta between 2 and 4 h after the start of feeding and maximum feed intake in the morning suggest fast transit times in turkeys. In chickens, excreta were sampled 3 and 6 h after provision of DON or DOM contaminated feed, so that transit times can only roughly be estimated. Still, maximum concentrations obtained at the latest 6 h after start of feeding corroborate fast excretion of DON- and DOM metabolites also in chickens.

Low concentrations of DON-3-sulfate and DON in chyme samples (content of jejunum and ileum) of pullets support the hypothesis of rapid and extensive absorption of DON from the small intestine [39]. The origin of DON-3-sulfate in jejunum and ileum might be biliary excretion of DON-3-sulfate formed in enterocytes or in the liver. As chyme samples were collected 3 h after administration of DON, biliary excretion might not have arrived at its maximum which was reported to occur *ca.* 6 h after dosing [37].

Presence of DOM-3-sulfate in excreta of pullets and roosters might be due to formation of DOM by intestinal microbes, absorption of DOM from the GI tract, sulfation in the intestinal mucosa, liver and/or kidney, and excretion into the cloaca via urine or back into the GI tract via bile. The latter could also explain low concentrations of DOM-3-sulfate in ileum. An additional hypothesis is antiperistaltic retrograde movement of DON-3-sulfate from the cloaca to the microbial rich caecae and microbe-mediated formation of DOM-3-sulfate from DON-3-sulfate. A third possibility might be sulfation in DOM by intestinal microbes. Absence of DOM-3-sulfate in excreta samples of our turkey and chicken trial might be due to differences in the intestinal microflora, specifically due to lack of microbes capable of de-epoxidizing DON. Although infection with *A. galli* resulted in significantly lower formation of DON-3-sulfate and DOM-3-sulfate in pullets, the formation pattern of DON metabolites was unaltered by worm infection.

Concluding from the DON sulfonate pattern across the GI tract of pullets, DON sulfonates are formed early in the digestion process. The mechanism of DON sulfonate formation is currently unknown. Some experiments aiming at elucidating the mechanism of DON-, DOM-, and DON-3-glucoside (D3G) sulfonate formation in rats had been carried out lately [24], but were inconclusive. However, because the major part of the sulfonates was recovered in feces of DON treated rats and the formation pattern shifted to DOM sulfonate on the second day after treatment, authors speculated that DON-, DOM-, and D3G sulfonates might be formed in a Michael addition with reactive inorganic sulfur species possibly present in intestinal chyme rather than by liver enzymes.

Biological recoveries of DON in the current study are much higher than those previously published for native DON administered to chickens, which were ≤5% [28]. However, they compare nicely to values published for experiments with radiolabeled DON. Wan *et al.* [25], using radioisotope counting radio-HPLC, reported biological recoveries of 81%, 89% and 95% in the time span of 24, 48 and 72 h after oral administration of a single dose of radiolabeled DON. Likewise, Prelusky *et al.* [37] recovered 79%, 92% and 99% of radiolabeled DON by 24, 48 and 72 h after single bolus administration to white Leghorn hens. The finding that excretion is not complete 24 h after treatment explains incomplete biological recoveries in our feeding trials with turkeys and chickens. Lower biological recoveries in turkeys than in chickens hint at delayed excretion in turkeys compared to chickens. Recoveries greater than 100% in the pullet trial might be due to presence of DON-3-glucoside (D3G) in the Fusarium contaminated diet used in the animal experiment. As D3G was not determined in the administered feed, its presence and subsequent *in vivo* metabolization to DON- and DOM-3-sulfate cannot be excluded.

To summarize, our findings and data from the recent literature substantiate, complement, and extend results and hypotheses from earlier published articles on the absorption, metabolism, and excretion of DON in poultry. DON is, to a great extent, rapidly absorbed from the GI tract between crop and jejunum. After absorption, DON is extensively metabolized to DON-3-sulfate in the intestinal mucosa, the liver, and possibly in the kidney. DON-3-sulfate is rapidly and completely eliminated into bile by the liver and/or into urine by the kidney. DOM-3-sulfate is an important natural DON metabolite in pullets and roosters and might originate from sulfation of microbe-formed DOM by intestinal mucosa, liver, and/or kidney or from microbe mediated deepoxidation of urinary DON-3-sulfate transported into caecae by retrograde movement.

## 4. Experimental Section

### 4.1. Reagents

Methanol (MeOH, LC gradient grade) and formic acid (98%–100%, p.a.) were purchased from Merck (Darmstadt, Germany). Formic acid (LC-MS gradient grade) for LC-MS/MS was obtained from Sigma Aldrich (Vienna, Austria). Reagents for synthesis of DOM-sulfates were purchased from ABCR (Karlsruhe, Germany) and Sigma Aldrich (Vienna, Austria). In all experiments, ultra-pure water (delivered by a Purelab Ultra system (ELGA LabWater, Celle, Germany)) was used.

### 4.2. Synthesis, Purification and Characterization of DOM-3-Sulfate and DOM-15-Sulfate

DOM as the starting material for synthesis of DOM-3-sulfate and DOM-15-sulfate was produced from DON as described earlier [24] and purified by preparative chromatography. The preparative HPLC system and column were the same as described in [34]. Mobile phase A consisted of methanol/water/formic acid (10/89.9/0.1, v/v/v), mobile phase B of methanol/formic acid (99.9/0.1, v/v). Gradient elution started at 0% B from 0 to 1 min and continued with linear increase to 100% B from 1 to 6 min. After 1 min at 100% B the starting conditions were re-established from 7 to 7.1 min and the column was re-equilibrated at 0% B until 10 min. The injection volume was 700 μL, the flow rate 16 mL/min and DOM was detected by measurement of UV absorption at 220 nm. The collection window of DOM was 5.66–6.03 min.

The synthesis of DOM-3-sulfate and DOM-15-sulfate was carried out as described for DON [31] with minor modifications. In short, it included synthesis of protected intermediates (4.2.1) and subsequent deprotection (4.2.2) followed by purification by column chromatography. The progress of all reactions was monitored by thin layer chromatography (TLC) using silica gel 60 F_254_ TLC plates (Merck, Germany). All chromatograms were visualized using ceric ammonium molybdate/Hanessian’s stain in EtOH/sulphuric acid. Column chromatography was performed on silica gel 60 (40–63 μm) using a Sepacore™ Flash System (Büchi, Switzerland) or small glass columns.

#### 4.2.1. Synthesis of the Protected Intermediates

DOM (140.0 mg, 0.50 mmol, 1.0 equivalents (eq.)) was dissolved in 20 mL of dry dichloromethane (DCM), cooled to 0 °C, and 1,2-dimethylimidazole (144.0 mg, 1.50 mmol, 3.0 eq.) was added to the mixture. Finally, 2,3-dimethyl-1-((2,2,2-trichloroethoxy)sulfonyl)-1H-imidazolium trifluoromethanesulfonate (342.8 mg, 0.75 mmol, 1.5 eq.) was added and the reaction was allowed to reach room temperature overnight. TLC after 1 day showed nearly full conversion of the starting material. Hence, the reaction was directly subjected to column chromatography (hexane/ethyl acetate 3/1, v/v) upon which three protected intermediates (2,2,2-trichloroethyl-DOM-3-sulfate (71.0 mg, 29%), 2,2,2-trichloroethyl-DOM-15-sulfate (14 mg, 6%) and bis(2,2,2-trichloroethyl) DOM-3,15-disulfate (30.0 mg, 9%)) were obtained.

#### 4.2.2. Deprotection of Protected Intermediates

Deprotection of the protected intermediates was carried out as described in [31]. The starting material was dissolved in MeOH (1 mL/10 μmol). Ammonium formate (3 eq. for the monosulfates, 6 eq. for the disulfate) as well as Zn dust (9 and 18 eq., respectively) were added, and the reaction mixture was placed in an ultrasonic bath at room temperature. The progress of the reaction was monitored by TLC until substantial amounts of products were formed (20 to 120 min). After filtration through celite, the remaining residue was subjected to column chromatography to end up with the corresponding sulfates as ammonium salts. For this purpose, a mobile phase of DCM/MeOH/NH_4_OH (10/4/1, v/v/v) was used. The final products were dissolved in water, lyophilized, and finally obtained as a white powder.

#### 4.2.3. NMR Spectroscopy

^1^H and ^13^C spectra were recorded on a Bruker Avance DRX-400 MHz and a Bruker Avance III 600 MHz spectrometer (Bruker, Germany). Data were recorded and evaluated using TOPSPIN 1.3 and TOPSPIN 3.2 (Bruker Topspin). All chemical shifts are given in ppm relative to tetramethylsilane. The calibration was done using residual solvent signals. Multiplicities are abbreviated as s (singlet), d (doublet), t (triplet), q (quartet) and b (broad signal). Deuterated solvents were purchased from Eurisotop (Gif sur Yvette Cedex, Paris, France).

#### 4.2.4. LC-HR-MS/MS

LC-HR-MS(/MS) spectra were recorded on a 6550 iFunnel Q-TOF instrument coupled to a 1290 Infinity UHPLC system (both Agilent Technologies, Waldbronn, Germany). Chromatographic separation was carried out on a Zorbax Eclipse Plus C18 Rapid Resolution High Definition column (2.1 × 150 mm, 1.8 μm particle size, Agilent, Waldbronn, Germany) at a flow rate of 0.25 mL/min using gradient elution (0 min: 15% B, 6 min: 100% B, 7 min: 100% B, 7.1 min: 15% B, 9 min: 15% B). Mobile phase A was water/formic acid (99.9/0.1, v/v), mobile phase B MeOH/formic acid (99.9/0.1, v/v). Compounds were ionized by electrospray ionization in the negative mode and measured first in full scan and then in targeted MS/MS mode at a collision energy of 30 eV (both in the range from *m/z* 40-1000). Electrospray ionization was carried out at a gas temperature of 140 °C, drying gas flow of 14 L/min, nebulizer pressure of 35 psig, sheath gas temperature of 350 °C, and sheath gas flow of 11 L/min. The capillary voltage was 4500 V, the nozzle voltage 300 V. Data acquisition was achieved in the 2 GHz extended dynamic range mode.

### 4.3. Standards and Standard Solutions

Solid DON (purity >95%) as well as standard solutions of DON and DOM (both 50 mg/L in acetonitrile) were supplied by Romer Labs GmbH (Tulln, Austria). DON-3-sulfate and DON-15-sulfate (both 95% purity) were synthesized according to [31]. DOM, DOM-3-sulfate, and DOM-15-sulfate were produced as described above (4.2). DON sulfonates 1, 2, 3 and DOM sulfonate 2 were produced as described in [24].

A mixed standard solution containing 300 mg/L of DON-3-sulfate, DOM-3-sulfate, DON, DOM, DONS 1, DONS 2, DONS 3, and DOMS 2 was prepared in methanol/water/formic acid (20/79.9/0.1, v/v/v). This solution and several dilutions thereof were used for spiking experiments and the establishment of pure solvent and matrix-matched calibration functions.

### 4.4. Sample Preparation Methods for Determination of DON, DOM, and Their Sulfates in Excreta and GI Samples of Poultry

Extraction of DON, DOM and their sulfate- and sulfonate metabolites from excreta samples was carried out according to a procedure previously optimized for the extraction of DON- and DOM sulfonates from rat feces [24]. A 300 mg aliquot of homogenized lyophilized excreta or GI content sample was consecutively extracted with 4, 3 and 3 mL of methanol/water/formic acid (49.5/49.5/1, v/v/v) by shaking for 30, 20 and 10 min in 15 mL polypropylene tubes. Prior to LC-MS/MS analysis, aliquots of the pooled extracts were diluted with water (turkeys, chickens: 1 + 2; pullets: jejunum and ileum: 1 + 1, excreta: 1 + 5; roosters: 1 + 9) and centrifuged at 14,000 × *g*.

### 4.5. LC-MS/MS Analysis of DON, DOM, Their Sulfates and Their Sulfonates in Excreta of Poultry

LC-MS/MS analyses were carried out on an Agilent 1290 series UHPLC system coupled to a 6500 QTrap mass spectrometer equipped with an IonDrive Turbo V^®^ source (Sciex, Foster City, CA, USA). Chromatographic separation was achieved on a Kinetex Biphenyl column (150 × 3 mm, 2.6 μm) protected by a SecurityGuard ULTRA pre-column of the same stationary phase (both Phenomenex, Aschaffenburg, Germany) at 30 °C and at a flow rate of 0.4 mL/min. Mobile phase A consisted of water/formic acid, mobile phase B of methanol/formic acid (both 99.9/0.1, v/v). Two different gradient methods were used, a short one for routine measurements, and a long one for separation of the 3/15 isomers of DON- and DOM-sulfate. The gradient of the short routine method was: 0.0–0.5 min: 10% B, 6.0 min: 90% B, 6.1–7.5 min: 100% B, 7.6–10.0 min: 10% B. Time segments of the gradient of the long method were: 0.0–0.5 min: 10% B, 4.5 min: 40% B, 8.0 min: 45% B, 8.5–10.9 min: 100% B, 11.0–13.5 min: 10% B. The injection volume was 3 μL and the LC eluent was diverted to the MS between 2.3 and 6.0 min (8.0 min for the long method).

Tandem mass spectrometric detection was performed in negative selected reaction monitoring (SRM) mode after electrospray ionization. The following ion source settings were used: Temperature 400 °C, ion spray voltage -4500 V, curtain gas 35 psi, ion source gas 1 80 psi, ion source gas 2 90 psi, collision gas (N_2_) high. SRM parameters (declustering potential, collision energy, collision cell exit potential) were optimized for the individual analytes by software-controlled compound optimization and are listed in Supplementary Table S3. Analyst^®^ software version 1.6.2 (Sciex) was used for instrument control and data evaluation.

### 4.6. Method Validation

Validation of the short routine method included determination of the apparent recovery (R_A_), recovery of extraction (R_E_), and mass spectrometric matrix effects (SSE) for excreta of turkeys and chickens as well as assessment of SSE for extracts of excreta and GI content samples of pullets, and excreta extracts of roosters. In addition, intra- and inter-day repeatability of sample work-up and analysis, limits of detection and quantitation, and linear range of calibration functions were evaluated. The long gradient method was validated with respect to SSE in excreta extracts of turkeys and chickens.

For assessment of R_A_, R_E_, and SSE in excreta samples of turkeys and chickens, 300 mg aliquots of freeze dried excreta collected before administration of toxins were spiked with 60 μL aliquots of spiking solutions containing between 0.5 and 300 mg/L of DON-3-sulfate, DOM-3-sulfate, DON, DOM, DONS 1, DONS 2, DONS 3, and DOMS 2, resulting in 0.1 to 60 mg/kg of these analytes in the freeze dried sample aliquots. Spiking was performed at seven concentration levels (0.1/0.3/1/3/10/30/60 mg/kg) in triplicate. One hour after spiking, 21 spiked and three unspiked samples were worked-up and diluted as described above, and measured in the same run as matrix-matched and pure solvent calibration functions. Pooled diluted extracts of unspiked samples were used to prepare matrix-matched calibration functions as described in detail in [24]. Both matrix-matched and pure solvent calibration functions were established at seven concentration levels (1.1/3.4/11/34/112/337/674 ng/mL), which corresponded to the theoretical analyte concentrations in measurement solutions of samples spiked prior to work-up in the case of 100% apparent recovery. R_A_, R_E_, and SSE were calculated by comparing the slopes of the standard addition curve (k_SA_), matrix-matched calibration curve (k_MM_), and pure solvent calibration curve (k_SOL_) as described in [41]. The following equations were used: R_A_ = k_SA_/k_SOL_ × 100; R_E_ = k_SA_/k_MM_ × 100; SSE = k_MM_/k_SOL_ × 100. Matrix-matched calibration functions were established between 3 and 600 ng/mL in diluted extracts of chyme and excreta samples of the negative control group of pullets and in excreta sample extracts of roosters, and used for calculation of SSE in these matrices. Apparent recoveries in the same matrices were estimated by multiplying the SSE of each individual analyte by the average R_E_ of the same analyte determined in excreta of turkeys and chickens (see above).

The intra-day repeatability was determined by triplicate work-up and measurement of blank turkey and chicken excreta samples spiked at four concentration levels (1, 3, 10, 30 ng/g) on one day. The inter-day repeatability was assessed by work-up and analysis of two randomly selected excreta samples (one of turkey, one of chicken) on each day sample preparation and analysis was carried out.

Limits of detection (LOD, signal to noise ratio (S/N) 3/1) and quantitation (LOQ, S/N 10/1) were determined in pure solvent standard solutions and in matrix-matched standard solutions. LODs and LOQs in freeze-dried samples were calculated by dividing LODs and LOQs in matrix-matched solutions by the recovery of extraction and by multiplying by the dilution factor. The linear range in matrix-matched calibration curves defined the upper end of the working range.

### 4.7. Design of the Feeding Trials

In total, excreta samples from four different feeding trials with poultry were analyzed. The parameters of the individual feeding trials are summarized in Table 5. Turkeys, chickens, and pullets of the negative control groups were fed with basal poultry feed naturally contaminated with 0.2–0.3 mg/kg DON. Turkeys and chickens of the DON group received basal poultry feed enriched with DON from culture material to a concentration of 1.5–1.7 mg/kg. Similarly, turkeys and chickens of the DOM group were fed with basal poultry feed artificially contaminated with the equimolar concentration of DOM. Pullets were exposed to 4.4 mg/kg DON in feed. As this feed was prepared from wheat contaminated with different Fusarium toxins [28], it also contained traces of 3-acetyl-DON (0.13 mg/kg) and 15-acetyl-DON (0.03 mg/kg). Roosters received diet containing 11 mg/kg DON.

All feeding trials and animal experiments were conducted following the European Guidelines for the Care and Use of Animals for Research Purpose [42]. The feeding trials with turkeys and chickens lasted for one day during which animals had unlimited access to feed and water until feed was removed in the evening. Animals were housed under a light/dark cycle of 18/6 h. Feed consumption of the individual animals was recorded. Excreta samples were collected at regular intervals during the day and in the morning of the following day. After the experiment, animals received basal poultry feed for two weeks before they were reintegrated into the meat production process. In the animal experiment with pullets, pullets received 65 g feed/day in two equal portions for two weeks and excreta samples were collected twice a day in the second week [28]. Pooled excreta samples were used for determination of biological recoveries. On the last day of the trial, pullets were slaughtered 3 h after the last feeding and chyme samples (contents of jejunum and ileum) were taken. Roosters were fed the DON contaminated feed at amounts of 90 g/day and excreta were collected for seven days [30]. Samples taken on different days were pooled for each animal prior to analysis. All excreta and chyme samples were stored frozen until lyophilization. Freeze-dried samples were stored at −20 °C.

**Table 5:** Overview of four DON/DOM feeding trials with poultry.

	Turkeys	Chickens	Pullets	Roosters
Reference	-	-	[28]	[29,30]
Animals	Hybrid Converter	Ross 308	Lohmann LSL	New Hampshire hybrids
Age	11 weeks	5 weeks	12 weeks	adult
No. of animals/group	4	4	9	8
c (DON) in feed (DON group) (mg/kg)	1.5	1.7	4.4	11
c (DON, DOM) in feed (DOM group) (mg/kg)	DON: 0.3 DOM: 1.4	DON: 0.2 DOM: 1.6	-	-
c (DON) in feed negative control group (mg/kg)	0.3	0.2	0.2	No NC
Feeding	Ad libitum access from 7:00 to 22:00	Ad libitum access from 7:00 to 19:00	65 g feed/day (2 equal portions)	90 g feed /day
Duration	1 day	1 day	2 weeks	9 days *
Sampling times	Every 2 h until 15:00; 18:00; 22:00; 7:00 next day	Every 3 h until 19 h; 7:00 next day	Excreta: morning and afternoon for 1 week; chyme: 3 h after administration	Morning and afternoon for 1 week
Samples taken	Excreta	Excreta	Excreta, content of jejunum and ileum	Excreta

* The duration of the animal experiment was 10 months, but the period of restricted feeding lasted for nine days.

### 4.8. Analysis of Samples and Data Evaluation

Excreta and GI content samples were worked-up in duplicate, each pooled and diluted extract was measured once. Analyte concentrations in freeze-dried samples were determined on the basis of pure solvent calibration functions (peak area *versus* analyte concentration) established routinely between 1 and 300 ng/mL under consideration of the apparent recoveries. In case the concentrations determined by duplicate sample work-up and analysis differed by more than 20%, work-up and analysis was repeated in duplicate and the new values were taken for further data processing.

For determination of the biological recoveries of ingested DON and DOM in the trials with turkeys, chickens, and pullets, DON equivalent amounts of all DON- and DOM metabolites quantified in excreta were calculated and divided by the DON equivalent amounts of DON or DOM ingested in the corresponding time period (one day for turkeys and chickens, one week for pullets). Statistical evaluation of differences in metabolite concentrations in excreta samples of pullets with and without *A. galli* infection was performed by one-way analysis of variance in MS Excel 2013.

### 4.9. Toxicity Assessment of DOM-3-Sulfate and DOM-15-Sulfate

To determine the *in vitro* toxicities of DON, DOM, DOM-3-sulfate, and DOM-15-sulfate, an *in vitro* transcription/translation assay with rabbit reticulocyte lysate (Promega, Madison, WI, USA) was employed. Transcription/translation reactions were carried out according to the manufacturer’s instructions as described in [33] for wheat germ extract with two modifications. Firstly, animal ribosomes (rabbit reticulocyte lysate) were used instead of wheat ribosomes. Secondly, the reactions were stopped after 20 min instead of 30 min. All substances tested were dissolved in water prior to use. The concentrations of DON in the assay were between 0 and 10 μM, while the concentration range of DOM and DOM-sulfates was 0–500 μM. For each compound, three independent assays were performed on three different days using the same batch of the reticulocyte lysate. For determining the translation efficiency at different inhibitor concentrations, one single test reaction was performed for each inhibitor at each concentration level. In addition, a control reaction was carried out at each concentration level where water was used instead of the test substance. The luciferase activity determined for the control reaction was set to 100% (uninhibited reaction) and the readouts from the other reactions were related to this control. Statistical evaluation was performed with IBM SPSS Statistics software. Comparison of mean values was performed with a two-sided *T*-test with Welch correction (equal variances not assumed). Results were considered significant at *p* < 0.05.

## 5. Conclusions

An LC-MS/MS based method for quantitative determination of DON-3-sulfate, DOM-3-sulfate, DON, DOM, DON sulfonates 1, 2, 3, and DOM sulfonate 2 in excreta and chyme samples of poultry was developed and validated. Application of the method to excreta and chyme samples from four different feeding trials with turkeys, broiler chickens, pullets, and roosters confirmed DON-3-sulfate as major DON metabolite in all investigated poultry species. Orally administered DOM was equally extensively metabolized to its 3-sulfate metabolite. In addition, DOM-3-sulfate was shown to be an important natural DON metabolite in pullets and roosters, where it amounted to *ca.* 12% and 33%, respectively, of all the detected metabolites. Interestingly, pullets not infected with *A. galli* excreted significantly greater concentrations and greater amounts of DON-3-sulfate and DOM-3-sulfate than pullets infected with worms. DON sulfonates of the series 1, 2 and 3 were detected in chyme samples of broilers, albeit only at trace levels. Biological recoveries of orally administered DON in the form of DON-3-sulfate and DOM-3-sulfate in chickens were close to 100%, supporting the hypothesis of nearly quantitative conversion of DON to its 3-sulfate metabolites in chickens. Although turkeys showed very similar metabolization of DON and DOM as chickens, biological recoveries of orally administered DON and DOM were only between 50% and 70%, suggesting faster excretion of DON and DOM metabolites in chickens than in turkeys.

Similar to DON-3-sulfate, DOM-3-sulfate was much less toxic than DON on the ribosome. Although *in vitro* transcription/translation assays cannot predict all aspects of *in vivo* toxicity, greatly reduced toxicity on the ribosome, the molecular target of trichothecenes, and low susceptibility of poultry to DON indicates that sulfation serves as detoxification mechanism for DON in poultry. Concluding from literature reports and our current study, locations of formation of DON-3-sulfate are most likely the intestinal mucosa, liver, and possibly the kidney. Elimination into excreta is probably achieved via bile and via urine. DOM-3-sulfate might also be formed by microbial conversion of DON-3-sulfate after retrograde movement of urinary DON-3-sulfate into the microbial rich caecae. In order to confirm this hypothesis, more complex animal experiments with separate collection of blood (hepatic and renal portal vein blood, systemic blood), bile, urine, contents of jejunum, ileum, caecae, and cloaca samples are required which demands highly sophisticated methods of sampling.

## Supplementary Materials

Supplementary materials can be accessed at: http://www.mdpi.com/2072-6651/7/11/4706/s1.
