# Supplementary material for: Metabolism of Deoxynivalenol and Deepoxy-Deoxynivalenol in Broiler Chickens, Pullets, Roosters and Turkeys
Source: Toxins (Basel). 2015 Nov 12;7(11):4706–29. doi: 10.3390/toxins7114706 (PMC4663529; doi:10.3390/toxins7114706)
Supplement: Supplementary file 1 [file toxins-07-04706-s001.pdf]

Supplementary Information

NMR and LC-HR-MS Data of Protected Intermediates:

NMR Data of 2,2,2-Trichloroethyl-DOM-3-Sulfate:

^{1}H NMR (400 MHz, $CDCl_3$) δ 6.57 (dq, *J* = 5.8, 1.6 Hz, 1H), 5.31 (s, 1H), 5.18 (s, 1H), 5.07 (dt, *J* = 11.9, 4.6 Hz, 1H), 4.82 (d, *J* = 10.9 Hz, 1H), 4.79 (d, *J* = 10.9 Hz, 1H), 4.68 (d, *J* = 5.9 Hz, 1H), 4.66 (s, 1H), 4.57 (d, *J* = 4.3 Hz, 1H), 3.80 (s, *2*H), 3.75 (s, 1H), 2.75 (dd, *J* = 15.6, 4.3 Hz, 1H), 2.09 (dd, *J* = 15.6, 11.6 Hz, 1H), 1.96 (br, 1H) 1.88 (s, 3H), 1.46 (s, 3H);
^{13}C-NMR (100 MHz, $CDCl_3$) δ 200.3 (s, 1C), 149.7 (s, 1C), 138.2 (d, 1C), 136.1 (s, 1C), 110 (t, 1C), 92.8 (s, 1C), 81.0 (d, 1C), 79.9 (t, 1C), 79.1 (d, 1C), 74.4 (d, 1C), 70.1 (d, 1C), 62.0 (t, 1C), 52.3 (s, 1C), 48.6 (s, 1C), 40.8 (t, 1C), 19.6 (q, 1C), 15.5 (q, 1C); LC-HR-MS *m/z* calcd for $C_{17}H_{20}O_8SCl_3^-$ $[M - H^+]^-$, 488.9950, found 488.9948.

NMR data of 2,2,2-Trichloroethyl-DOM-15-Sulfate:

^{1}H NMR (400 MHz, $CDCl_3$) δ 6.67 (dq, *J* = 5.9, 1.6 Hz, 1H), 5.26 (s, 1H), 5.10 (s, 1H), 4.94 (d, *J* = 5.9 Hz, 1H), 4.72 (s, 1H), 4.62 (d, *J* = 10.1 Hz, 1H), 4.61 (d *J* = 10.9 Hz, 1H), 4.56 (d, *J* = 10.9 Hz; 1H), 4.45 (d, *J* = 10.5 Hz, 1H), 4.30 (dt, *J* = 10.7, 4.2 Hz, 1H), 4.26 (d, *J* = 4.3 Hz, 1H), 3.78 (s, 1H), 2.20 (dd, *J* = 15.0, 4.1 Hz, 1H), 1.94–2.06 (m, 2H), 1.89 (s, 3H), 1.37 (s, 3H);
^{13}C-NMR (100 MHz, $CDCl_3$) δ 199.9 (s, 1C), 151.1 (s, 1C), 139.1 (d, 1C), 136.0 (s, 1C), 109.8 (t, 1C), 92.6 (s, 1C), 81.2 (d, 1C), 79.8 (t, 1C), 73.1 (d, 1C), 72.2 (t, 1C), 69.5 (d, 1C), 69.3 (d, 1C), 51.8 (s, 1C), 49.0 (s, 1C), 44.3 (t, 1C), 19.4 (q, 1C), 15.5 (q, 1C); LC-HR-MS *m/z* calcd for $C_{17}H_{20}O_8SCl_3^-$ $[M - H^+]^-$, 488.9950, found 488.9942.

NMR Data of Bis (2,2,2-Trichloroethyl) DOM-3,15-Disulfate:

^{1}H NMR (400 MHz, $CDCl_3$) δ 6.65 (dq, *J* = 5.6, 1.6 Hz, 1H), 5.39 (s, 1H), 5.24 (s, 1H), 5.09 (dt, *J* = 11.5, 4.4 Hz, 1H), 4.81 (s, 2H), 4.77 (d, *J* = 5.9 Hz, 1H), 4.70 (s, 1H), 4.61–4.66 (m, *2*H), 4.59 (d, *J* = 10.5 Hz, 1H), 4.54 (d, *J* = 10.5 Hz, 1H), 4.47 (d, *J* = 10.5 Hz, 1H), 3.76 (br, 1H), 2.58 (dd, *J* = 16.0, 4.3 Hz, 1H), 2.18 (dd, *J* = 15.6, 11.3 Hz, 1H), 1.91 (s, 3H), 1.43 (s, 3H);
^{13}C-NMR (100 MHz, $CDCl_3$) δ 199.3 (s, 1C), 148.3 (s, 1C), 138.3 (d, 1C), 136.4 (s, 1C), 112.0 (t, 1C), 92.7 (s, 1C), 92.5 (s, 1C), 80.2 (t, 1C), 80.0 (t, 1C), 79.8 (d, 1C), 78.9 (d, 1C), 73.0 (d, 1C), 71.8 (t, 1C), 69.3 (d, 1C), 51.6 (s, 1C), 48.4 (s, 1C), 40.9 (t, 1C), 19.1 (q, 1C), 15.4 (q, 1C); LC-HR-MS *m/z* calcd for $C_{19}H_{21}O_{11}S_2Cl_6^-$ $[M - H^+]^-$, 698.8662, found 698.8681.

NMR and LC-HR-MS Data of DOM-Sulfates:

NMR Data of DOM-3-Sulfate, Ammonium Salt:

^{1}H NMR (400 MHz, methanol-*d_4*) δ 6.59 (dq, *J* = 5.9, 1.6 Hz, 1H), 5.21 (s, 1H), 5.04 (s, 1H), 4.94–4.80 (m, 7H), 4.68 (dt, *J* = 11.3, 4.5 Hz, 1H), 4.60 (s, 1H), 4.41 (d, *J* = 4.3 Hz, 1H), 3.76 (d, *J* = 12.1 Hz, 1H), 3.72 (d, *J* = 12.1 Hz, 1H), 2.73 (dd, *J* = 15.0, 4.5 Hz, 1H), 1.93 (dd, *J* = 15.0, 11.5 Hz, 1H), 1.81 (q, *J* = 0.8 Hz, 3H), 1.42 (s, 3H); ^{13}C NMR (100 MHz, methanol-*d_4*) δ 202.3 (s, 1C), 153.9 (s, 1C), 139.6 (d, 1C), 136.9 (s, 1C), 108.3 (t, 1C), 81.5 (d, 1C), 75.7 (d, 1C), 75.6 (d, 1C), 71.7

(d, 1C), 61.7 (t, 1C), 54.1 (s, 1C), 49.5 (s, 1C), 43.3 (t, 1C), 20.1 (q, 1C), 15.4 (q, 1C); LC-HR-MS: *m/z* calculated for $C_{15}H_{19}O_8S^-$ $[M - H^+]^-$: 359.0806, *m/z* measured: 359.0807.

NMR Data of DOM-15-Sulfate, Ammonium Salt:

^{1}H NMR (400 MHz, methanol-d_4) δ 6.62 (dq, J = 6.0, 1.5 Hz, 1H), 5.16 (s, 1H), 5.02 (d, J = 6.2 Hz, 1H), 5.01 (s, 1H), 4.95–4.80 (s, NH_4^+, 2 x CH, H_2O), 4.70 (s, 1H), 4.20 (d, J = 10.5 Hz, 1H), 4.16 (s, 1H), 4.14 (dt, J = 15.2, 4.5 Hz, 1H), 4.01 (d, 10.9 Hz, 1H), 2.56 (dd, J = 14.6, 4.1 Hz, 1H), 1.88 (dd, J = 15.0, 10.7 Hz, 1H), 1.83 (s, 3H), 1.43 (s, 3H); ^{13}C NMR (100 MHz, methanol-d_4) δ 201.7 (s, 1C), 154.7 (s, 1C), 140.1 (d, 1C), 136.9 (s, 1C), 107.8 (t, 1C), 82.8 (d, 1C), 76.0 (d, 1C), 70.9 (d, 1C), 70.1 (d, 1C), 67.1 (t, 1C), 52.8 (s, 1C), 50.2 (s, 1C), 45.7 (t, 1C), 20.0 (q, 1C), 15.4 (q, 1C); LC-HR-MS: *m/z* calculated for $C_{15}H_{19}O_8S^-$ $[M - H^+]^-$: 359.0806, *m/z* measured: 359.0806.

NMR Data of DOM-3,15-Disulfate, Diammonium Salt:

^{1}H NMR (600 MHz, methanol-d_4) δ 6.64 (dq, J = 5.7, 1.5 Hz, 1H), 5.23 (s, 1H), 5.07 (s, 1H), 5.05 – 4.80 (m, 2 x NH_4^+ + 1H + H_2O), 4.72–4.60 (m, 3H), 4.45 (d, J = 4.4 Hz, 1H), 4.23 (d, J = 10.9 Hz, 1H), 4.03 (d, J = 10.9 Hz, 1H), 2.81 (dd, J = 15.3, 4.4 Hz, 1H), 1.99 (dd, J = 15.3, 11.4 Hz, 1H), 1.83 (s, 3H), 1.46 (s, 3H); ^{13}C NMR (150 MHz, methanol-d_4) δ 201.6 (s, 1C), 153.6 (s, 1C), 139.9 (d, 1C), 137.1 (s, 1C), 108.6 (t, 1C), 81.5 (d, 1C), 75.7 (d, 1C), 75.5 (d, 1C), 71.0 (d, 1C), 67.1 (t, 1C), 52.9 (s, 1C), 49.1 (s, 1C), 43.4 (t, 1C), 19.9 (q, 1C), 15.5 (q, 1C); LC-HR-MS: *m/z* calculated for $C_{15}H_{19}O_{11}S_2^-$ $[M - H^+]^-$: 439.0374, *m/z* measured: 439.0375.

Table S1. Matrix effects of the long gradient method. Dilution of extracts: 1 + 2, v + v.

	Average ± standard deviation (n = 3)									
	DON-3-sulfate	DON-15-sulfate	DOM-3-sulfate	DOM-15-Sulfate	DON	DOM	DONS 1	DONS 2	DONS 3	DOMS 2
Excreta of turkey	87 ± 3	95 ± 1	89 ± 5	103 ± 2	89 ± 1	90 ± 1	89 ± 4	105 ± 5	101 ± 5	108 ± 0
Excreta of broiler (1)	105 ± 2	135 ± 0	104 ± 1	114 ± 2	107 ± 2	107 ± 2	108 ± 1	119 ± 4	188 ± 4	117 ± 1

Table S2. Calculation of biological recoveries in the pullet experiment. -: no *A. galli* infection, +: *A.galli* infection.

	Amount of Lyophilized Excreta (g)	µg Excreted Over 7 Days (in DON Equivalents)						
		DON-3-sulfate	DOM-3-sulfate	DON	DONS 2	SUM	µg DON Ingested	Biological Recovery (%)
DON -	109 ± 3	2496 ± 104	379 ± 20	84 ± 3	16 ± 1	2975 ± 121	1973 ± 23	151 ± 5
DON +	110 ± 9	2227 ± 182	272 ± 113	85 ± 7	14 ± 5	2598 ± 214	1981 ± 0	131 ± 11

Table S3. Optimized SRM parameters.

Analyte	Retention Time (min)		Precursor Ion (m/z)	Ion Spezies	DP (V)	Product Ions (quant/qual) (m/z)	CE (eV)	Relative Intensity (qual/quant)
	Short Method	Long Method						
DONS 1	2.60	2.76	377.1	$[M-H]^-$	−130	80.0/331.0	−98/−52	0.25
DONS 2	3.40	3.81	377.1	$[M-H]^-$	−105	81.0/347.0	−68/−36	0.21
DONS 3	3.92	4.66	377.1	$[M-H]^-$	−125	347.0/80.0	−36/−98	0.29
DOMS 2	4.19	5.10	361.1	$[M-H]^-$	−25	81.0/249.0	−65/−30	0.51
DON-3-sulfate	4.43	5.58	375.1	$[M-H]^-$	−125	344.9/247.0	−36/−38	0.58
DON-15-sulfate	4.38	5.46	375.1	$[M-H]^-$	−110	97.0/229.0	−38/−42	0.20
DOM-3-sulfate	4.74	6.18	359.1	$[M-H]^-$	−125	97.0/329.0	−38/−34	0.65
DOM-15-sulfate	4.80	6.28	359.1	$[M-H]^-$	−125	97.0/80.0	−28/−118	0.26
DON	4.95	6.43	341.1	$[M+HCO_2]^-$	−25	265.1/45.0	−14/−38	1.01
DOM	5.44	7.50	325.1	$[M+HCO_2]^-$	−25	45.0/249.1	−40/−17	0.32

(**A**) ^{1}H spectrum of 2,2,2-trichloroethyl-DOM-3-sulfate

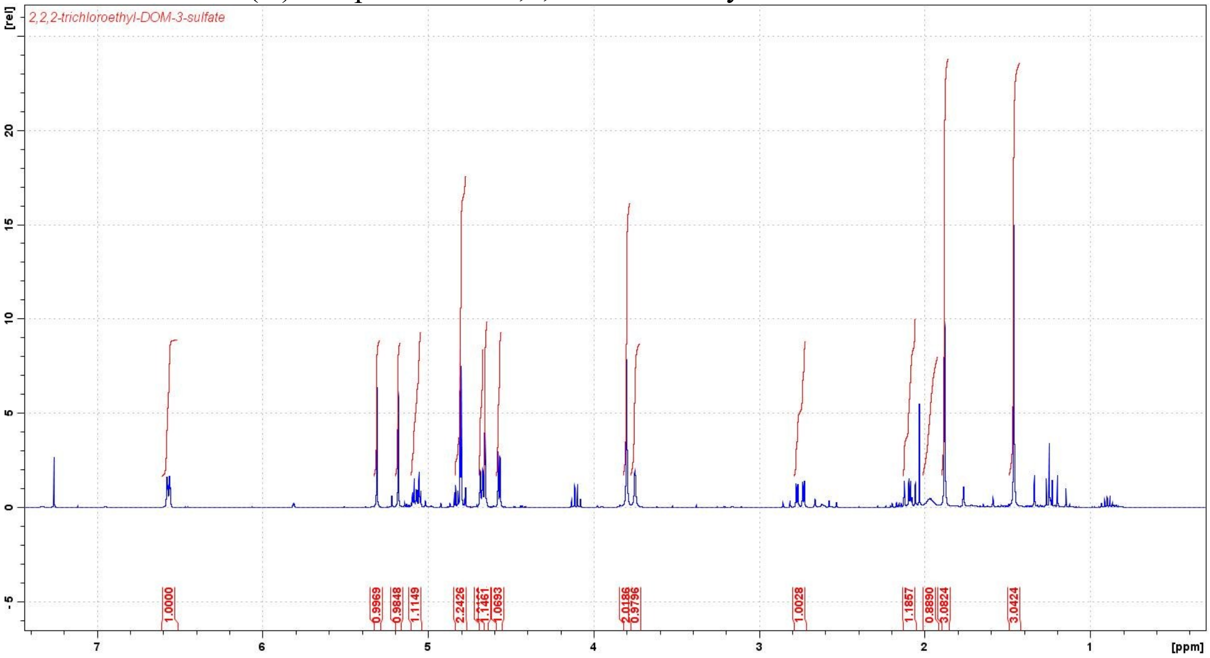

(**B**) ^{13}C spectrum of 2,2,2-trichloroethyl-DOM-3-sulfate

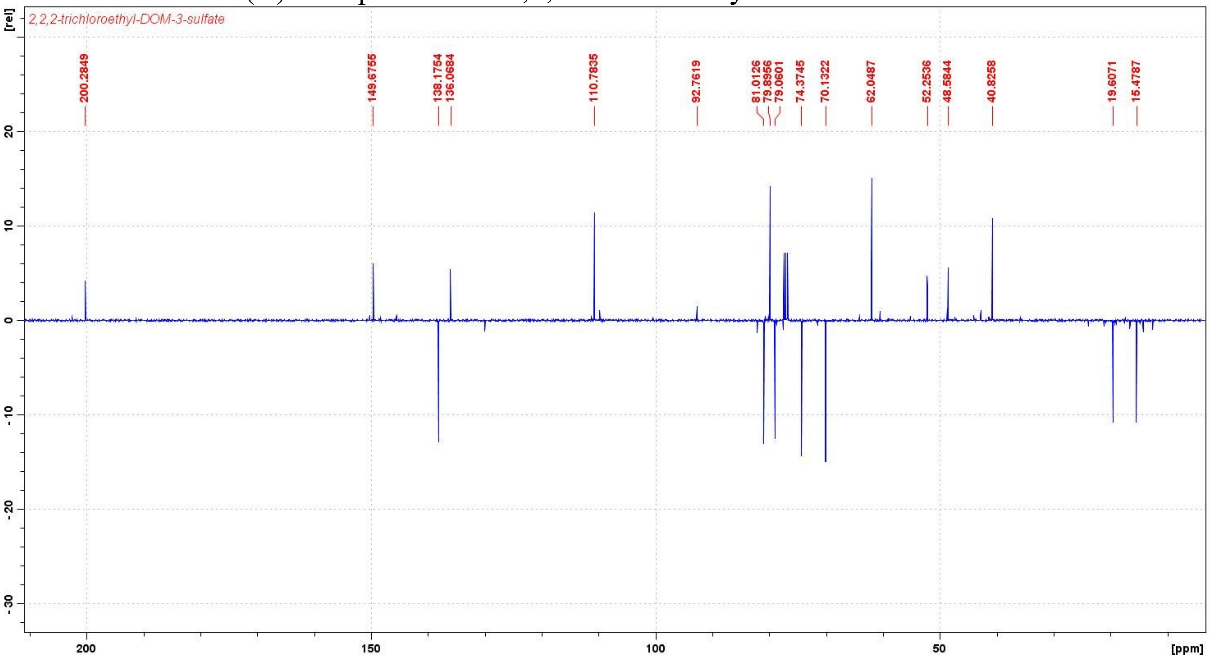

Figure S1. *Cont.*

(**C**) ^{1}H spectrum of 2,2,2-trichloroethyl-DOM-15-sulfate

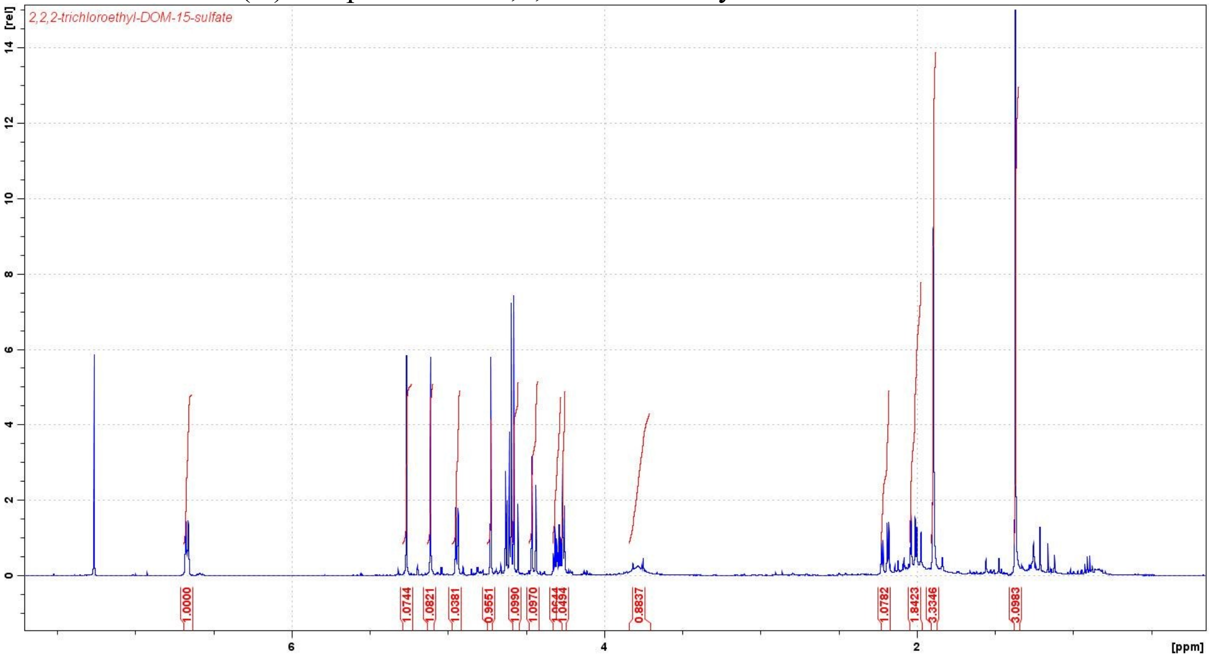

(**D**) ^{13}C spectrum of 2,2,2-trichloroethyl-DOM-15-sulfate

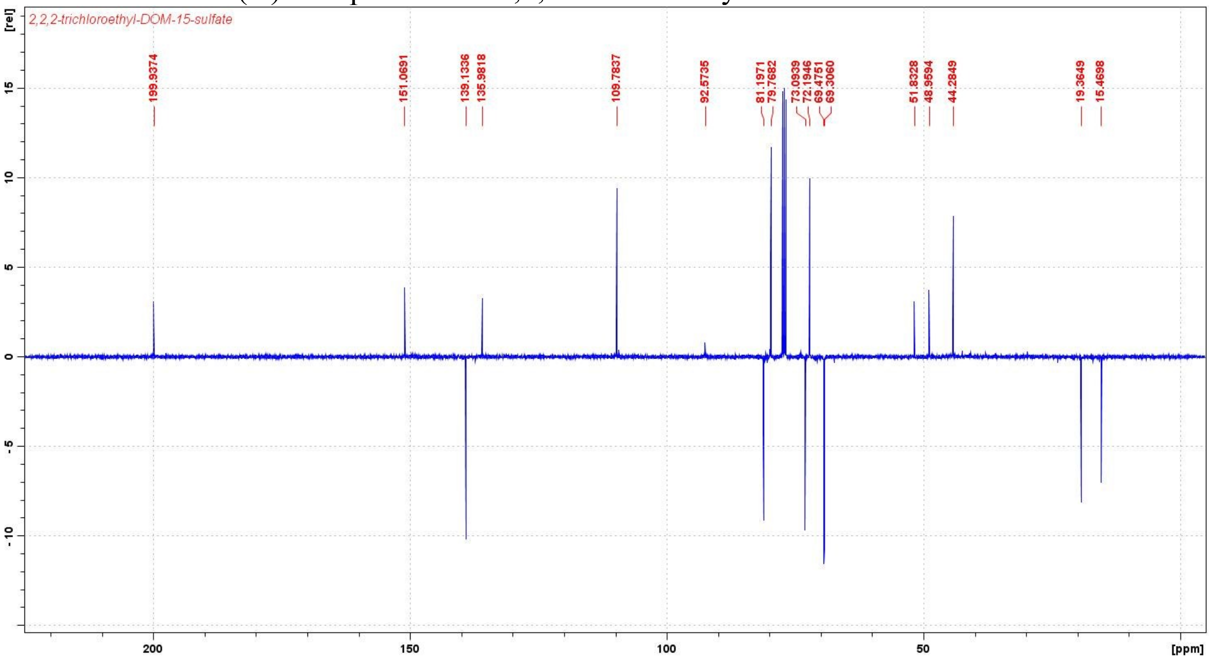

Figure S1. *Cont.*

(**E**) ^{1}H spectrum of bis(2,2,2-trichloroethyl) DOM-3,15-disulfate

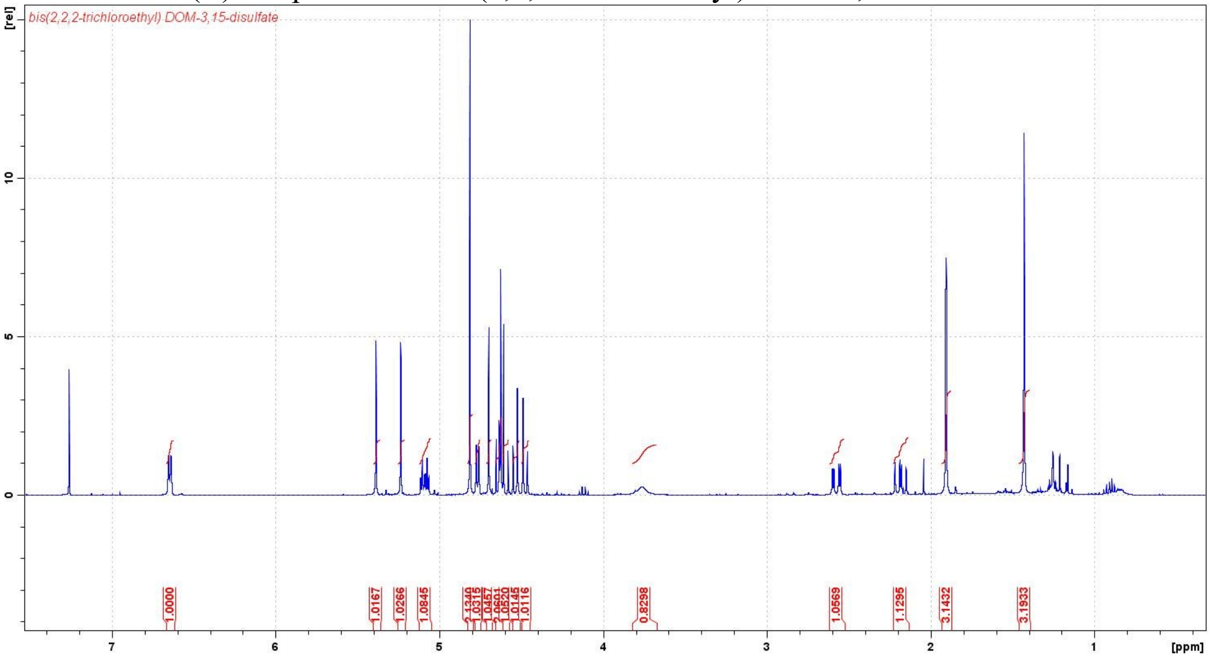

(**F**) ^{13}C spectrum of bis(2,2,2-trichloroethyl) DOM-3,15-disulfate

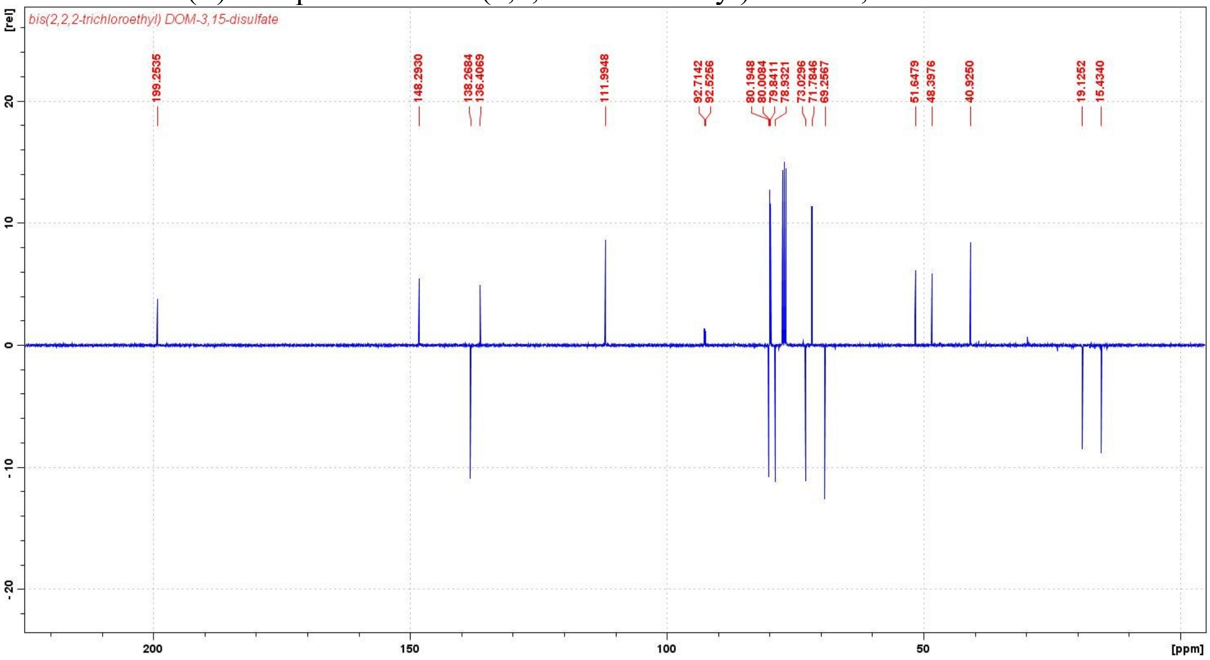

Figure S1. NMR spectra of protected intermediates.

(**A**) ^{1}H spectrum of DOM-3-sulfate, ammonium salt

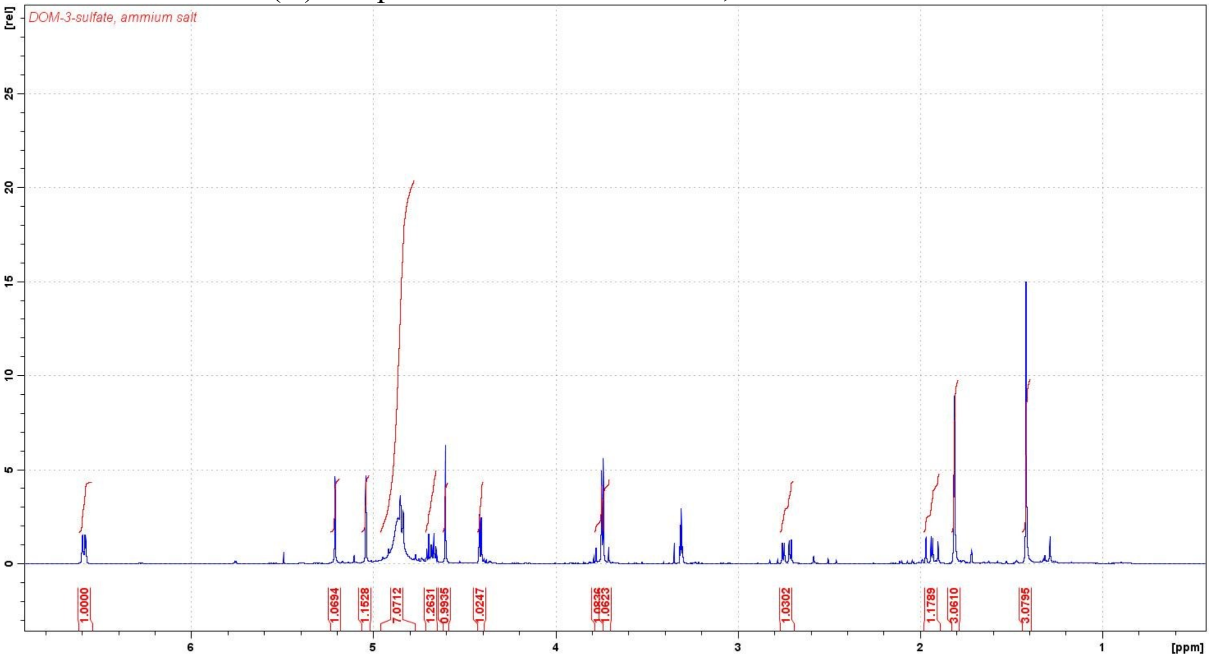

(**B**) ^{13}C spectrum of DOM-3-sulfate, ammonium salt

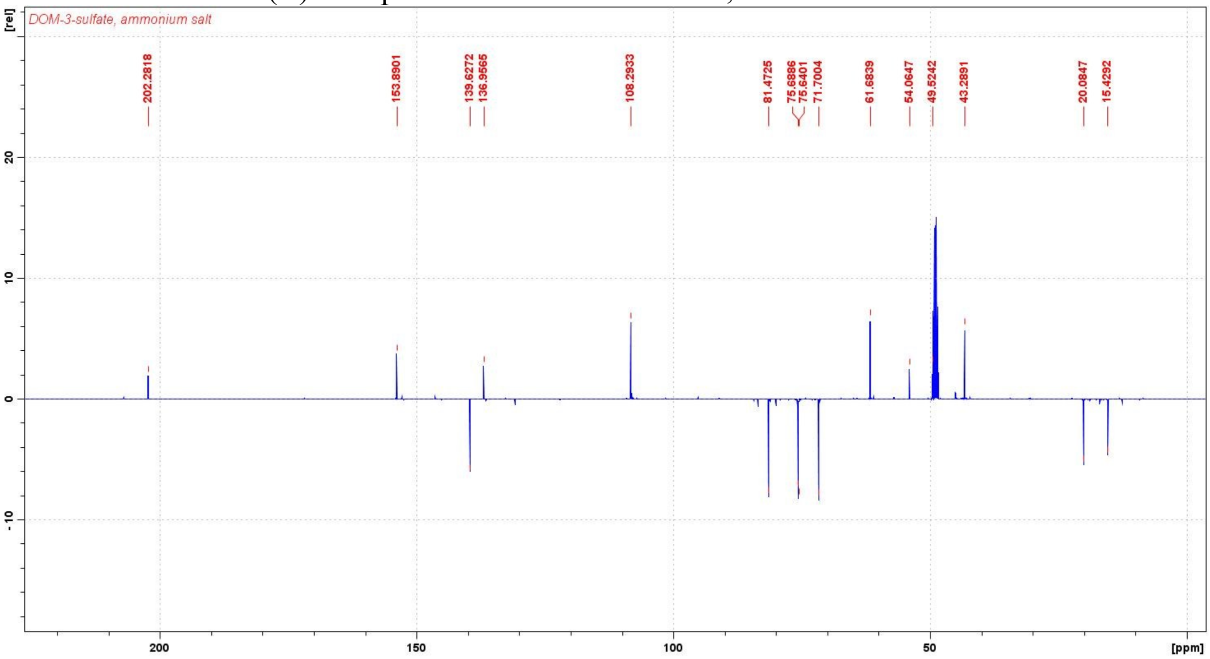

Figure S2. *Cont.*

(**C**) ^{1}H spectrum of DOM-15-sulfate, ammonium salt

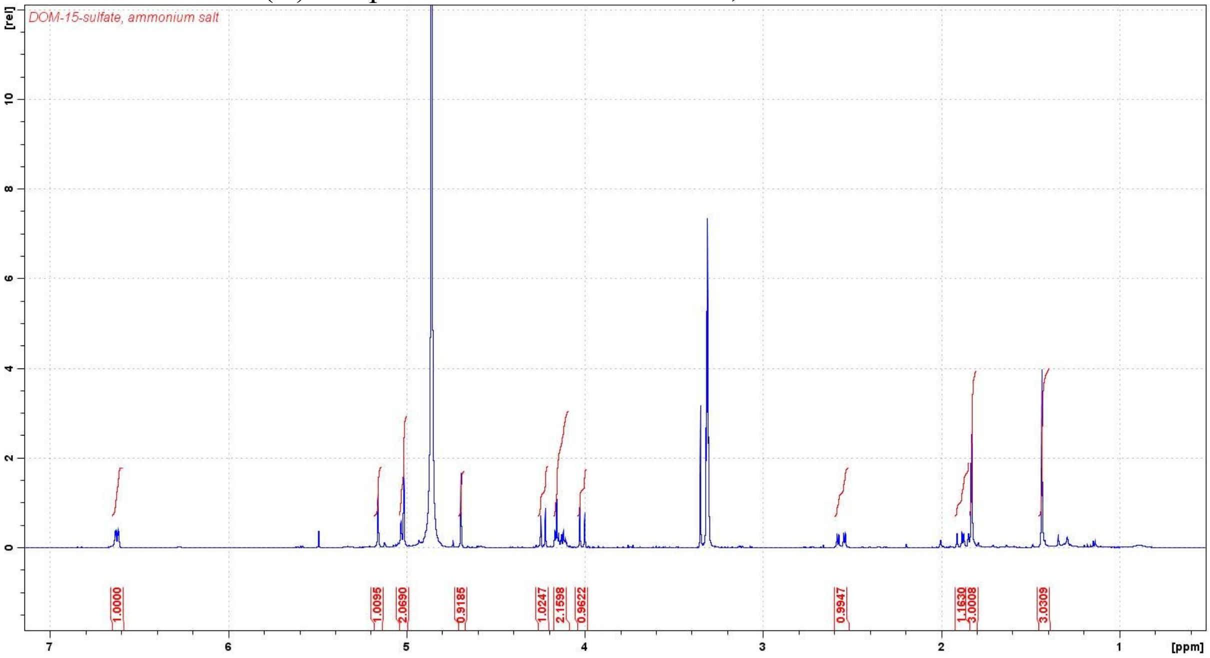

(**D**) ^{13}C spectrum of DOM-15-sulfate, ammonium salt

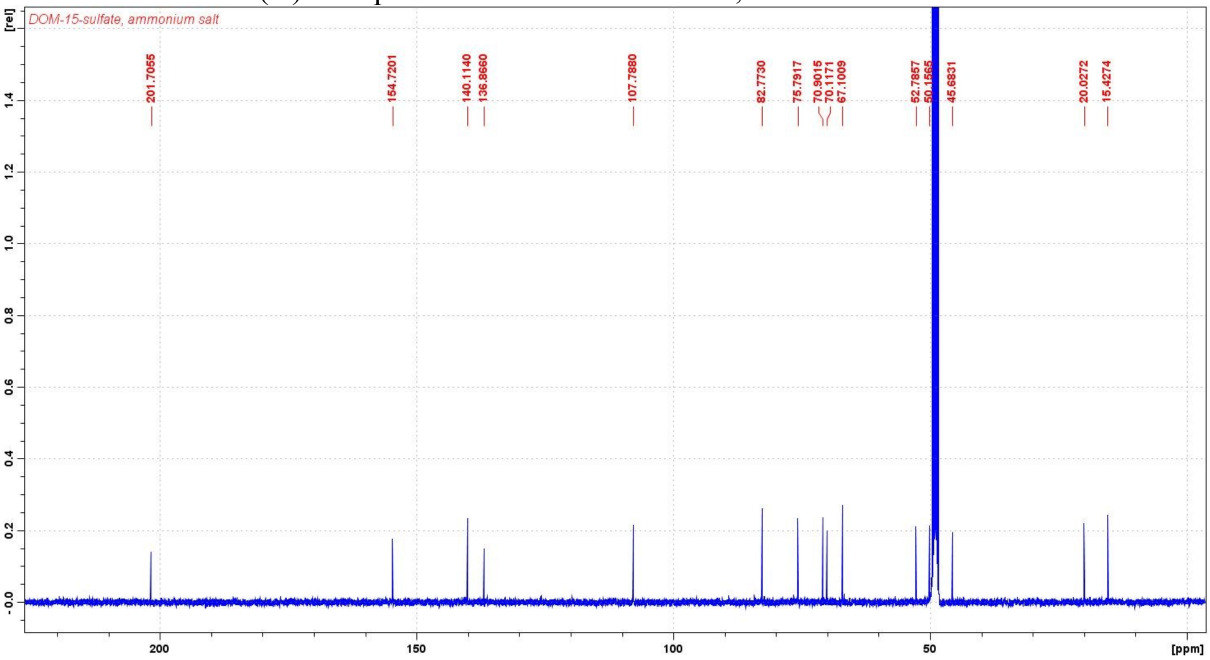

Figure S2. *Cont.*

(**E**) ^{1}H spectrum of DOM-3,15-disulfate, diammonium salt

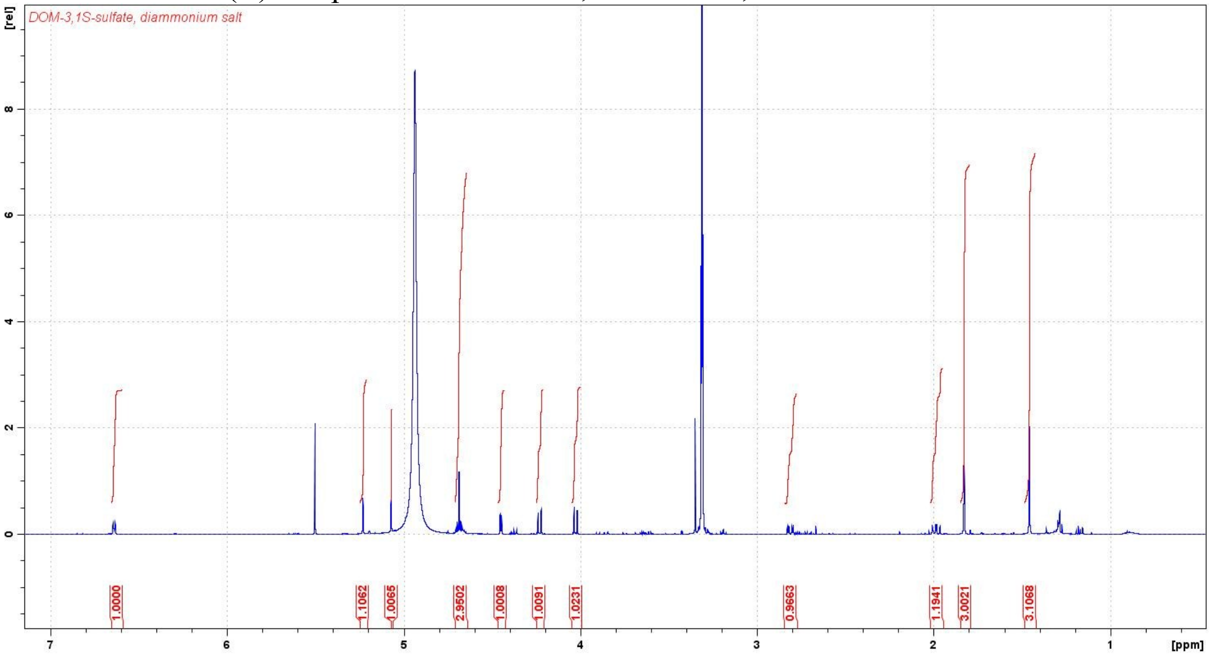

(**F**) ^{13}C spectrum of DOM-3,15-disulfate, diammonium salt

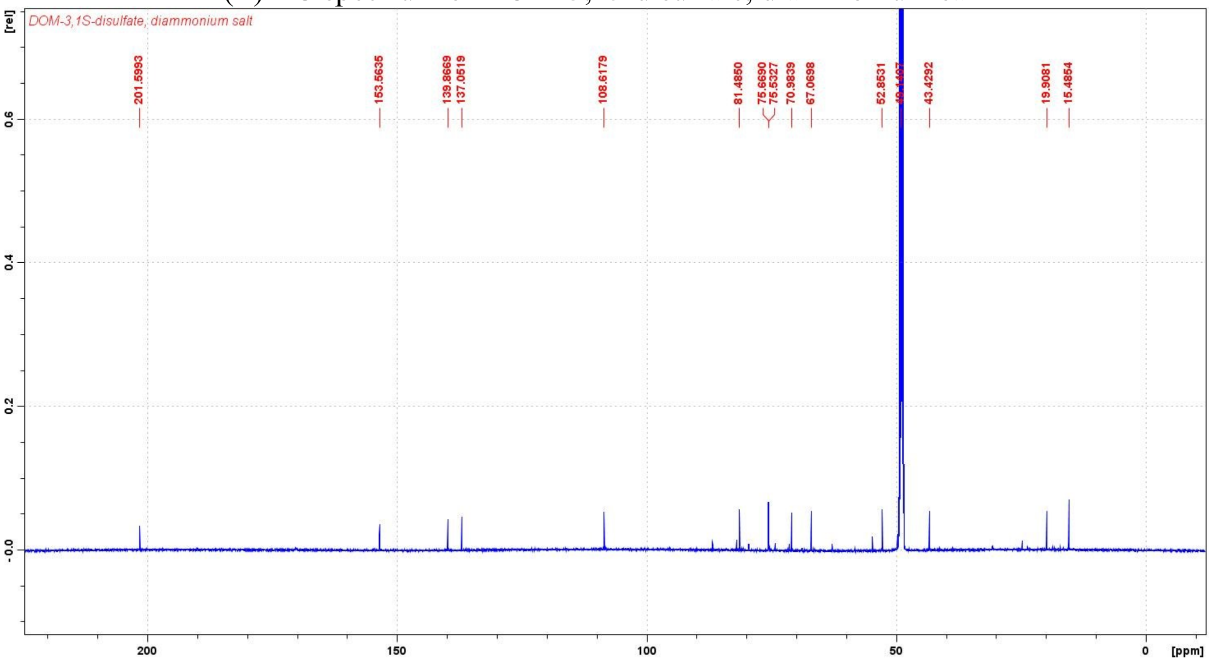

Figure S2. NMR Spectra of DOM-sulfates (ammonium salts).

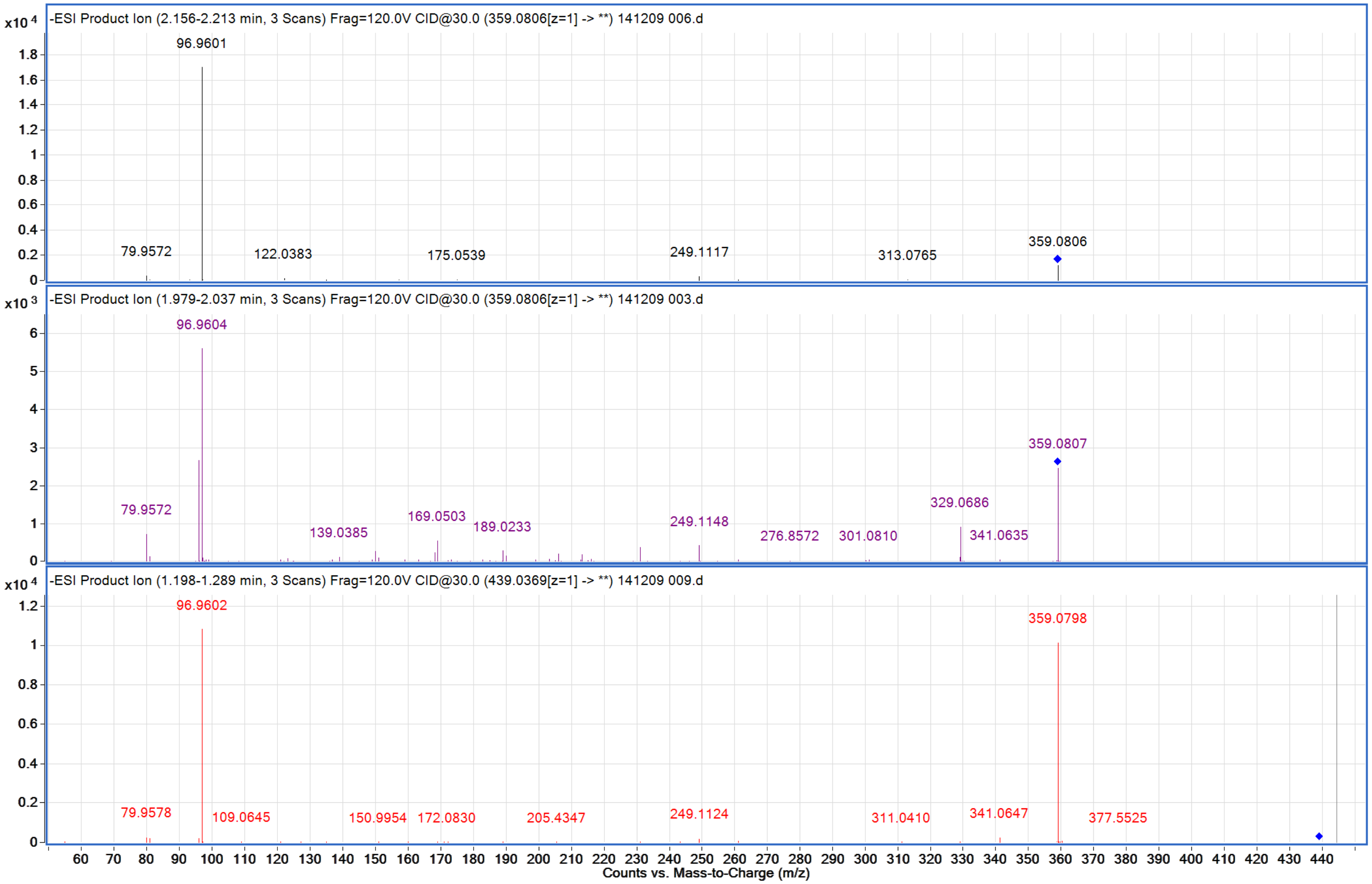

Figure S3. HR-MS/MS spectra of DOM-3-sulfate, DOM-15-sulfate and DOM-3,15-disulfate (CE 30 eV).

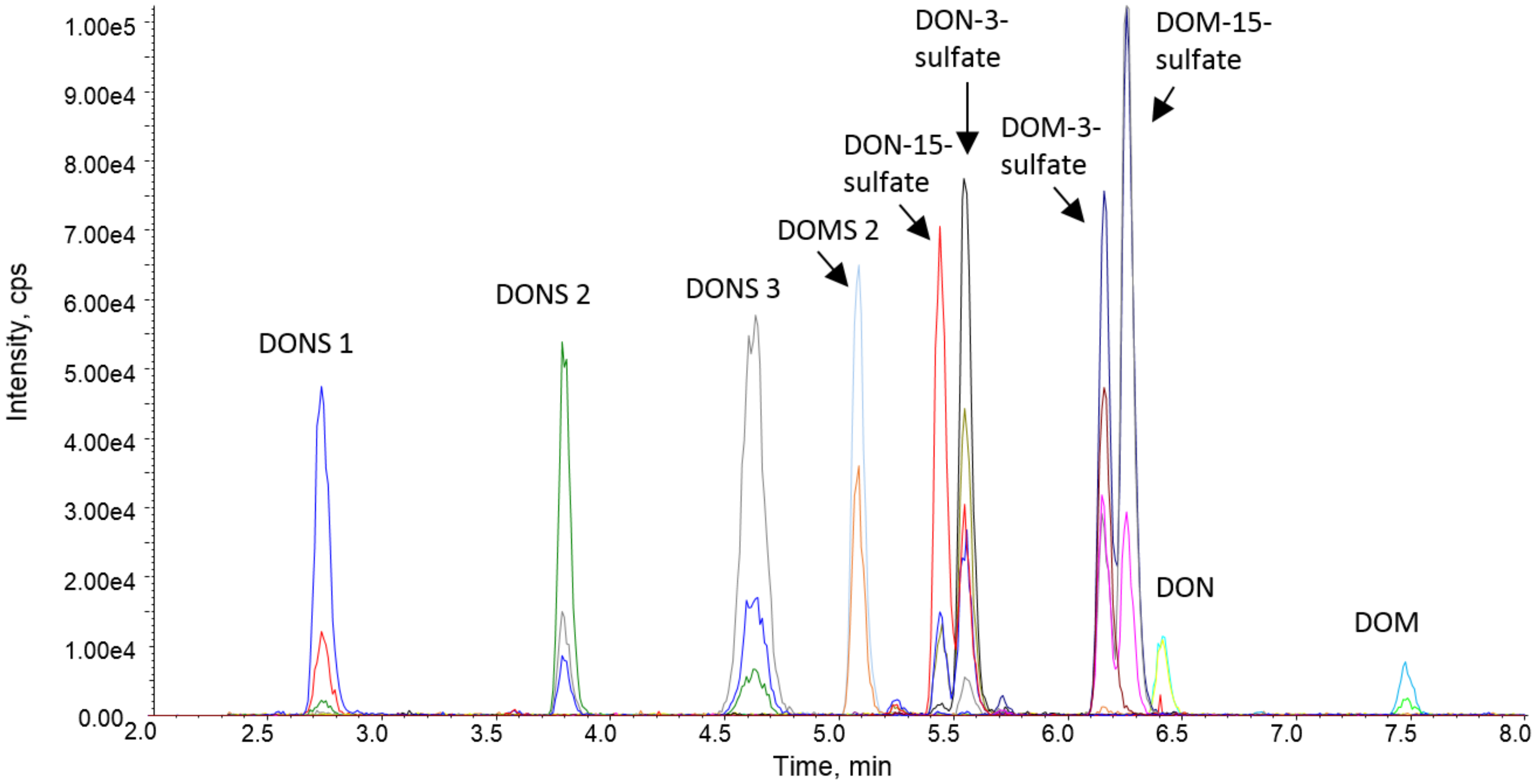

Figure S4. LC-MS/MS chromatogram of a standard solution containing 30 ng/mL of all analytes (long gradient method).
